# Insight into Hypoxia Stemness Control

**DOI:** 10.3390/cells10082161

**Published:** 2021-08-22

**Authors:** Miriam Di Mattia, Annunziata Mauro, Maria Rita Citeroni, Beatrice Dufrusine, Alessia Peserico, Valentina Russo, Paolo Berardinelli, Enrico Dainese, Annamaria Cimini, Barbara Barboni

**Affiliations:** 1 Unit of Basic and Applied Biosciences, Faculty of Bioscience and Agro-Food and Environmental Technology, University of Teramo, 64100 Teramo, Italy; mdimattia@unite.it (M.D.M.); mrciteroni@unite.it (M.R.C.); apeserico@unite.it (A.P.); vrusso@unite.it (V.R.); pberardinelli@unite.it (P.B.); edainese@unite.it (E.D.); bbarboni@unite.it (B.B.); 2 Department of Innovative Technologies in Medicine & Dentistry, University of Chieti-Pescara, 66100 Chieti, Italy; bdufrusine@unite.it; 3 Center of Advanced Studies and Technology (CAST), 66100 Chieti, Italy; 4 Department of Life, Health and Environmental Sciences, University of L’Aquila, 67100 L’Aquila, Italy; annamaria.cimini@univaq.it; 5 Sbarro Institute for Cancer Research and Molecular Medicine and Center for Biotechnology, Temple University, Philadelphia, PA 19122, USA

**Keywords:** hypoxia, O_2_ tension, hypoxia inducible factors, intracellular signaling, metabolism, stemness, hypoxia in vitro models

## Abstract

Recently, the research on stemness and multilineage differentiation mechanisms has greatly increased its value due to the potential therapeutic impact of stem cell-based approaches. Stem cells modulate their self-renewing and differentiation capacities in response to endogenous and/or extrinsic factors that can control stem cell fate. One key factor controlling stem cell phenotype is oxygen (O_2_). Several pieces of evidence demonstrated that the complexity of reproducing O_2_ physiological tensions and gradients in culture is responsible for defective stem cell behavior in vitro and after transplantation. This evidence is still worsened by considering that stem cells are conventionally incubated under non-physiological air O_2_ tension (21%). Therefore, the study of mechanisms and signaling activated at lower O_2_ tension, such as those existing under native microenvironments (referred to as hypoxia), represent an effective strategy to define if O_2_ is essential in preserving naïve stemness potential as well as in modulating their differentiation. Starting from this premise, the goal of the present review is to report the status of the art about the link existing between hypoxia and stemness providing insight into the factors/molecules involved, to design targeted strategies that, recapitulating naïve O_2_ signals, enable towards the therapeutic use of stem cell for tissue engineering and regenerative medicine.

## 1. Introduction

### 1.1. The Role of O_2_ in Cell Biology

Molecular oxygen (O_2_) is necessary for animal life and is essential for a variety of biological processes involved in the survival of prokaryotic and eukaryotic cells. The rate of O_2_ usage by cells is various, depending on the cell type and function. In eukaryotic cells, O_2_ uptake occurs by direct transport across the cell membrane and 90% of O_2_ is consumed by mitochondria during respiration and oxidative phosphorylation processes [1]. Furthermore, the citric acid cycle and β-oxidation of fatty acids are tightly associated with the process of ATP production. Thus, O_2_ availability is essential for cell functions, and decreased O_2_ concentration represents a major stress factor for cells. In a homeostatic state, cells require a level of O_2_ between 2–9% (14.4–64.8 mmHg), lower levels of O_2_ in cells are related to a state of hypoxia 0.5–2% (<10 mmHg) [2]. Cells modulate gene expression in response to O_2_ availability and these changes affect cell metabolism, immunity, and tissue reorganization [3]. Cellular adaptive responses to hypoxia are mainly mediated by the transcription factor hypoxia-inducible factor-1 (HIF-1) which induces transcriptional activation of various genes promoting angiogenesis, cell proliferation, and survival in hypoxic conditions. Cells activate multiple adaptative responses for O_2_ supply: (I) reduce the rate of oxidative phosphorylation, (II) arrest cell cycle, (III) stimulate the formation of new blood vessels by releasing the major angiogenic factors (vascular endothelial growth factor (VEGF), angiopoietin1 (Ang-1), transforming growth factor β1 (TGF-β1), and fibroblast growth factor (FGF-2)), and (IV) switch to anaerobic glycolysis [4]. Furthermore, morphological cytoskeletal cellular changes occurring in hypoxia, such as alteration in protein polarization and aggregation, lead to an increase in membrane permeability [5]. O_2_ dyshomeostasis, such as high O_2_ levels, can also induce cytotoxicity due to the production of reactive O_2_ species (ROS) during its utilization. ROS include peroxides, singlet O_2_, hydroxy radical, and superoxide which are responsible for lipid, protein, and nucleic acids oxidation causing cellular dysfunction. Cells have different levels of antioxidants and redox enzymes to contrast the ROS accumulation. Unfortunately, these defense systems are not always adequate to contrast ROS production resulting in different levels of ROS tolerance.

### 1.2. The Role of O_2_ in Tissue

**Figure 1:**
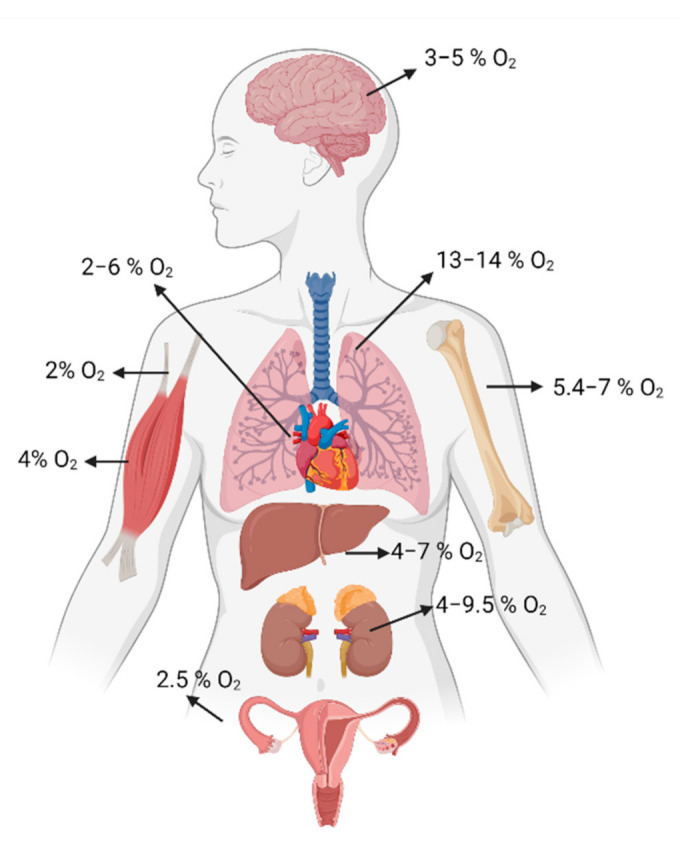
Different O_2_ partial pressure in body districts.

Within an organism O_2_ is up taken in the lungs, it passes into the alveoli and by simple diffusion across endothelial cells of the alveolar capillaries. Once in the circulation, O_2_ is transported into the blood in two forms; mainly bound with hemoglobin or dissolved in plasma [6]. The O_2_ tension of inspired air is 160 mmHg, in alveolar blood is 104 mmHg while in most tissues is around 40–50 mmHg [7,8] (Figure 1). However, in several tissues O_2_ level is lower such as in the spleen, thymus, retina, and regions of the brain where it has been measured around 16, 10, 25, and 8 mmHg [8]. More in detail, low O_2_ levels have been associated to various stem cell niches, such as mesenchymal stem cells (MSCs), neural stem cells (NSCs), and hematopoietic stem cells (HSCs), suggesting a pivotal role of O_2_ in maintaining stem cells pluripotency as discussed along this review. All tissues have their own characteristic “tissue-normoxia” and oxygen dyshomeostasis induces damage depending on the tissue affected. Furthermore, O_2_ levels vary in tissues during normal physiological states such as skeletal muscle exertion or embryo development. However, the hypoxic state in tissues is characteristic of pathological conditions that occur in infection, ischemic cardiovascular disease, chronic obstructive pulmonary disease, or cancer [2,9,10,11,12]. For example, oxygenation is very low in various areas of many solid tumors due to the uncontrolled proliferation of cells and abnormal blood vessels spreading. Moreover, hypoxic tissues are induced by impaired vascular function characterizing tissue wound. In the wound healing process, macrophages accumulate preferentially in hypoxic niches where respond rapidly by activating an array of adaptive genes [13]. Adaption of macrophages alters the expression of receptors and protein adhesion to further enhance their migration towards hypoxic sites. Furthermore, hypoxia-induced macrophages release growth factors and cytokines to recruit mesenchymal cells involved even in early wound healing events [13]. Hypoxia occurs in the later phases of reepithelization and restoration of tissue integrity and vasculature [14]. Furthermore, considering the heterogenicity of the cell populations characterizing each tissue niche it would be necessary to understand interactions between multiple cell types in hypoxic microenvironments and to investigate the response mechanisms to increased O_2_ levels.

### 1.3. Hypoxia-Inducible Factor (HIF)

Hypoxia-inducible factor (HIF) is the master regulator of O_2_ homeostasis with hundreds of hypoxia-inducible target genes. HIF is a heterodimeric transcription factor consisting of two subunits: HIF-α and HIF-β [15]. HIF-β is considered a constitutively expressed gene while HIF-1α is the predominant regulator of hypoxia and it is mainly regulated at post-translational level [16]. Nowadays, three HIF-α subunits (HIF-1α, HIF-2α, and HIF-3α) have been recognized [17], with different functions [18], exhibiting high conservation of the protein domain structures and regulation of the hypoxia-dependent mechanisms. The three HIF isoforms differing in the oxygen-sensitive α subunit exist in vertebrates [19]. All the three isoforms form the heterodimer with HIF-1β binding to the same cis-element HIF-binding sites (HBS) [20].

HIF-1α and HIF-2α (EPAS1) are structurally similar and best characterized. HIF-3α (IPAS) exists as multiple splice variants, able to inhibit HIF-1α and HIF-2α activity [21]. HIF-1α is expressed in all cells, while HIF-2α and HIF-3α are selectively expressed in vascular endothelial cells, type II pneumocytes, renal interstitial cells, liver parenchymal cells, and cells of the myeloid lineage [22]. Even if they display similar biochemical properties, distinct physiological roles of HIF-1α and HIF-2α can be supposed [20]. It was demonstrated that embryos HIF-1α^–/–^ nor HIF-2α^–/–^ did not survive, suggesting that HIF-1α and HIF-2α have different and not complementary functions [20].

HIF is normally expressed in cells at the basal level, but in the presence of high an unphysiological air levels of O_2_, 21% O_2_ concentration generally defined as “normoxia”, it is ubiquitinated and degraded. In detail, the DNA-binding domain of HIF-1α lies within the N-terminal region of the protein, whereas the *C*-terminal region holds the two transactivation domains. The central region of HIF-1α contains an O_2_-dependent degradation (ODD) domain, located between the amino acids 401 and 603, which confers O_2_-sensitiviy to the HIF-1α subunit [23]. Its conserved proline residues are hydroxylated by prolyl hydroxylase domain enzymes (PHDs) creating a binding site for the von Hippel–Lindau (VHL) protein, a component of the E3 ubiquitin ligase complex, which leads HIF-1α subunit to the proteasomal destruction [24]. As a result, HIF-1α is rapidly degraded in normoxic conditions. When low O_2_ concentration, as hypoxia occurs, PHDs are inactive, HIF-1α is not transcriptionally upregulated but the protein was stabilized.

**Figure 2:**
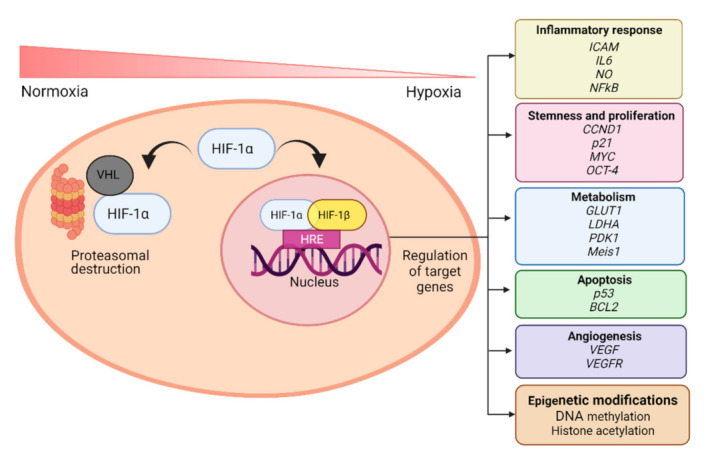
Scheme of HIF-1α activation during different O_2_ level exposure. Under normoxia condition (≅21% O_2_) HIF is rapidly degraded by proteasomal machinery. During hypoxia (≤10% O_2_) HIF is stabilized and translocated into the nucleus where heterodimerized with HIF-1β. Heterodimer HIF-1α/β, regulates HRE target genes (some of which are indicated) involved in different biological responses.

In a low O_2_ environment HIF-α and HIF-β subunits form a heterodimer creating the aryl hydrocarbon receptor nuclear translocator complex (ARNT), which translocates into the nucleus [23] (Figure 2). The ARNT complex formation is O_2_ concentration-dependent [18]. When the ARNT heterodimer is assembled in the nucleus, it could be recognized by the co-activator and could bind to the conserved consensus sequence 5′-(A/G)CGTG-3′ within the hypoxia-response elements (HRE) of O_2_-regulated target genes modulating transcription [15,25]. HIFs can induce the transcription of more than 70 genes correlated with control O_2_ homeostasis, angiogenesis, mitochondrial metabolism [25], and adaptive functions [26] including epigenetic DNA modification, mRNA, microRNA, and protein synthesis [4] related to different biological responses (Figure 2).

It has been indicated that HIF-1α and HIF-2α differ in their ability to transactivate hypoxia-inducible genes. Indeed, it was proved that some genes were transactivated exclusively by HIF-1α, such as genes coding for glycolytic enzymes, while others were transactivated by both [27]. By using siRNA interference, it was shown that a small group of genes having binding sites for the E-twenty-six (ETS) family of transcription factors in common, were regulated by HIF-2α. Knock-down of ELK-1, the most abundant member of ETS family, significantly reduced hypoxic induction of the HIF-2α—dependent genes [28]. HIF-2α is supposed to have a relevant role in angiogenesis since it specifically regulates VEGF receptor Flk-1 expression, even if the mechanism was not well elucidated [29].

HIF-1α and HIF-2α regulate, also, angiogenic *VEGF* genes [27,30,31]. In this context, it has been reported that, under a hypoxic environment, mutant mice with HIF-1α deletion in the endothelial stem cells (ECs) showed defective blood vessel growth and activation of VEGF and its receptor VEGFR2, accompanied by impaired cell proliferation and migration. The results obtained in the study lead the authors to hypothesize that HIF-1α induces an autocrine VEGF/VEGFR2 regulation in ECs promoting their functions in tissue angiogenesis [32].

Many studies have reported that HIF is involved in many pathways influencing, in particular, cells cycle, proliferation, metabolism, stem cells plasticity, angiogenesis, and immunomodulation [33] (Figure 2). HIF-1α can directly reprograms the metabolic state in cells. Both HIF-1α and HIF-2α can modulate the expression of cytochrome c oxidase isoforms and maximize the efficiency of the electron transport chain [34,35]. The deficit of this mechanism negatively affects the production of ATP and leads to a major ROS production in hypoxia. Moreover, HIF-1α encodes for the pyruvate dehydrogenase kinase 1 acting through the target gene PDK, which represses the flux of pyruvate into acetyl-CoA, suppressing O_2_ consumption [35,36]. In knock-out HIF-1α cells, hypoxia contributes to reduced ATP levels, elevated ROS, and apoptosis [34,36].

HIF also has an active role in inflammatory conditions as it promotes nuclear factor-κB (NF-κB) activity, a family of inducible transcription factors regulating a large array of genes involved in different processes of the immune and inflammatory responses [37] in macrophages, neutrophils, and nonimmune cells [38]. The NF-κB proteins are normally sequestered in the cytoplasm by a family of inhibitory proteins, including IκB family members. Hypoxia inhibits PHD1 activity resulting in IKK activation and phosphorylation of IκB followed by its degradation with the consequent liberation of NF-kB from the cytoplasm inducing the transcription of inflammatory cytokines [39].

Moreover, HIF can promote the expression of several miRNAs [40]. Out of all miRNAs influenced by HIF, miR-210 is the most significantly induced by hypoxia in all cell lines [41]. Its expression is regulated by both HIF-1α [42] and HIF-2α [43]. Overexpression of miR-210 in HUVECs leads to enhanced VEGFA and VEGFR2 expression promoting angiogenesis [44]. Interestingly, it was proposed that miR-210 could contribute to the HIF switch between HIF-1/HIF-2 and HIF-3 in human chondrocytes [45] and hepatocellular carcinoma cells [46] as miR-210 directly targets HIF-3α and suppresses HIF-1α protein expression.

Even if PHD is recognized as the main regulator of HIF-1α [47], different factors influence the ultimate result of the HIF activity, such as the presence or absence of HRE in gene promoter; the structure of variable sequences in HRE element of gene influencing the selective co-operation of other transcription factors, co-activators, or co-inhibitors with HIF; the cell type that present specific expression, compartmentalization, and degradation location of HIF-α isomers [48].

## 2. Hypoxia and Stemness

O_2_ concentration has been closely linked to the maintenance of stemness in stem cells that in vivo reside in specific “tissue niches”, the anatomic locations that regulate their participation in tissue generation, maintenance, and repair [49]. Stem cell niche is a complex, heterotypic, and dynamic structure which includes supporting extracellular matrix, neighboring niche cells, secreted soluble signaling factors, physical, and environmental signals [50,51]. Comprehensive studies to clarify their critical components have been performed and stem cell niche’s structures have been identified in many germlines and adult tissues [50,51]. It is known that hypoxia is recorded inside them and, even if the exact O_2_ inside the niches in vivo cannot be recorded with the currently techniques, based on the closest approximations performed in human, an average O_2_ of 3–13% O_2_ exist in stem cell niche [7] (Figure 1). By residing in these dynamic tightly controlled in vivo environments that experience relatively low O_2_ tensions, stem cells maintain a selective advantage suitable for their biological roles. Hence, reproducing the O_2_ tension existing in native microenvironments represents a major challenge for researchers that might exploit it as a good strategy to preserve or enhance stem cells features with the advantage of their therapeutic value in regenerative medicine.

This review aims to clarify the state of art concerning the link between hypoxia and stemness to investigate the heterogeneity and complexity of the biological cues influencing the native local signal of stem cells, as well as to compare the hypoxia strategies and related aspects for preservation and improvement of stem cells properties.

**Figure 3:**
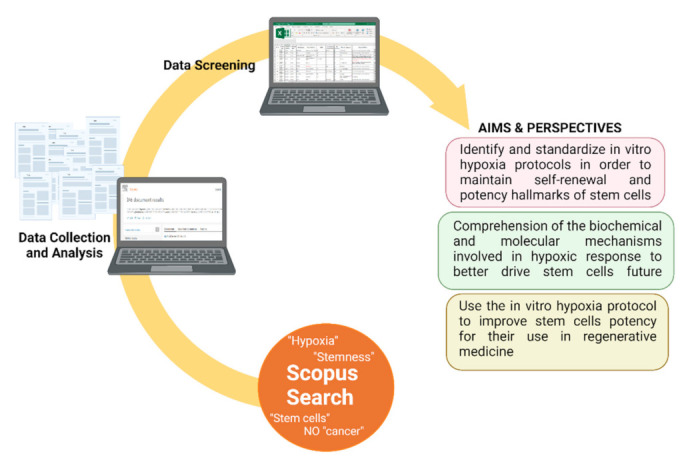
Schematic representation of methodology for data research and analysis performed with specific keywords in Scopus Database related to the link between hypoxia and stemness preservation elaborated in this review.

Bibliographic papers dealing with this topic present in Scopus Database have been analyzed by using specific keywords, among these “hypoxia”, “stemness”, and “stem cells”. To only select papers strictly related to the topic of the research, all articles associated with cancer stem cells and cancer progression were excluded (Figure 3).

Many in vivo and in vitro approaches aiming to mimic the naive hypoxia cellular microenvironment experienced to stem cells, have been reported. Animal models are usually employed to study the effect of in vivo hypoxia, especially to characterize a wide variety of diseases, including reoxygenation injury, pre-eclampsia, diabetic retinopathy, and hypoxic insult of the brain [52]. However, animal study findings are often characterized by a great biological variability such as that recorded for values related to minute ventilation, tidal volume, peripheral O_2_ saturation, arterial CO_2_ pressure, and exhaled NO levels [52,53,54]. In addition, hypoxia animal models in vivo fail to recapitulate some of the key hallmarks of stem cell physiology, leading researchers to approach hypoxia studies by using in vitro cell cultures. More efforts have been made to exploit the possibility to grow stem cells in in vitro hypoxic conditions to mimic the niche microenvironment. However, outside their hypoxic natural environment, stem cells undergo physiological changes inducing high variabilities in their therapeutic efficacy remaining an open challenge for researchers and clinicians. In this context, for example, the O_2_ gradient in culture conditions proposed for mesenchymal stem cells (MSCs) are different and, often, characterized by controversial results causing ambiguity in the interpretation of hypoxia effects. Main differences in methods, physical or chemical induction, O_2_ percentages or chemical compound concentrations, stem cells models, time of hypoxic exposure, and different modality of HIF activation were reported, making it difficult for the optimization of hypoxic induction protocols to be used. In addition, stem cell metabolism has recently emerged as a critical determinant of cellular processes and is uniquely adapted to support proliferation, stemness, and commitment. Metabolic activation is also linked to HIF factor, which transcriptionally activates genes involved in O_2_ homeostasis and metabolism [25,55]. Moreover, HIF-dependent mechanisms can influence many other processes as an epigenetic response including DNA methylation and histone acetylation, which in turn modulate hypoxia-responsive gene expression in cells. Nevertheless, HIF-1 pathway could be activated from stimuli different from hypoxic ones, as “hypoxic mimetic compounds”, showing the same final effect on stemness preservation [56] but increasing the variability of hypoxic methods that can be applied in vitro.

Data collected by our scientometric analysis revealed a complexity of factors involved in the strategies mostly used for hypoxic induction and in the correlation between hypoxia and stemness. The purposes of this review are:

- Define and standardize in vitro hypoxia protocols to maintain self-renewal and potency hallmarks of stem cells.
- Comprehend the biochemical and molecular mechanisms involved in the hypoxic response to better drive stem cell future.
- Use in vitro hypoxia protocols to improve stem cell potency for their use in regenerative medicine.

**Figure 4:**
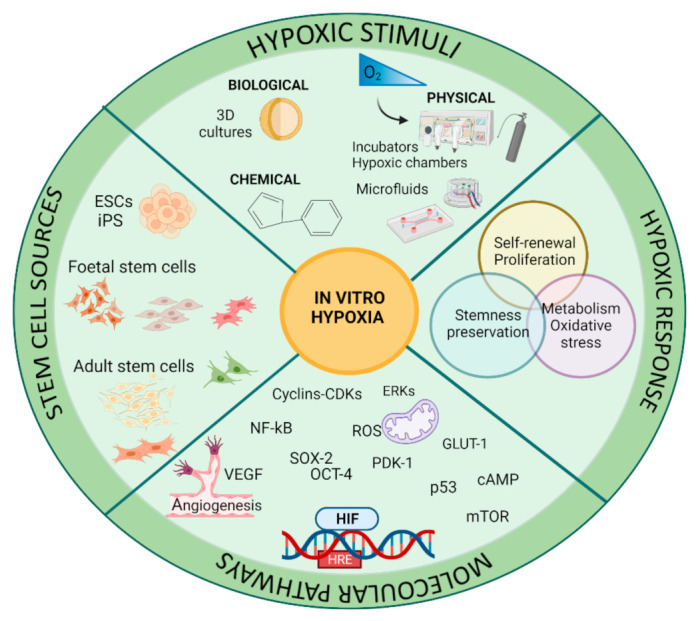
Parameters and outcomes for in vitro hypoxia exploitations. Different hypoxic stimuli, cells sources, biological responses, and pathways principally discussed in the review.

We will consider the in vitro stimuli for hypoxia (physical, chemical, and biological) describing hypoxia exploitation for regenerative medicine (O_2_ and HIF stemness preservation, stem metabolic state, and ROS), taking into account the most used stem cells sources (Figure 4).

## 3. In Vitro Models of Hypoxia

Hypoxia can be differently induced in vitro, and several elements must be considered, as physical or chemical induction, O_2_ percentages or chemical compound concentrations, and the time of exposure [57]. Cells can sense changes in the O_2_ tension, defined as the activity of dissolved molecules in their microenvironment, influencing growth and differentiation processes. Routinely, in vitro cell cultures are performed in liquid culture media incubated at the atmospheric O*_2_* concentration of 21%, and it is difficult to know the exact O_2_ tension that cells experience because it is strictly correlated to the rate of O_2_ consumption by the cells [58]. Recently, it has been reported that in laboratory practice exists a considerable number of parameters, often non cited in literature, that generate a wide variability in O_2_ delivery compromising results reproducibility. Culture dishes and their geometries, cell types, seeding density, media volume and its composition, culture temperature, and opening the doors of the incubator are frequently considered as principal factors influencing O_2_ delivery in cell cultures [59] (Table 1).

Frequent handlings of cell cultures imply exposing cells at the atmospheric O_2_ air tension where re-oxygenation rapidly occurs thus generating fluctuations in O_2_ concentration. This factor can affect, for example, the lysis of those proteins that are susceptible to rapid oxygenation-dependent modifications [60]. Furthermore, medium changes interrupt the concentration gradient that is established over time in cell cultures since culture medium requires significant amounts of time to equilibrate to new O_2_ concentrations [59]. This is a crucial aspect, and the improvement of the in vitro oxygenation control would be very advantageous to reach a functional in vivo resemblance [61].

Given the difficulties in controlling O_2_ levels, appropriate O_2_ sensors should be applied in cell cultures to measure O_2_ concentration thus monitoring its fluctuations, however, they are not routinely used [62].

The use of hypoxic chambers or incubators with a specific mixture of nitrogen gas (N_2_), carbon dioxide (CO_2_), and O_2_ represent a common approach to regulate O_2_ tension in in vitro culture. To achieve low O_2_ levels, more N_2_ is introduced in the gas mixture, thus reducing the partial pressure of O_2_. Alternatively, incubators can be connected to an external high-pressure liquid nitrogen tank that infuses N_2_ displacing O_2_. Among different methods adopted to this aim, the most innovative accredited system is the hypoxia workstation [60] that can offer precise control of O_2_ and CO_2_, as well as, control of temperature and relative humidity, providing to maintain a hypoxic environment in long-term cell culture. More details on O_2_ different induction in cells culture are reported in Section 3.1 of this review.

**Table 1:** Relevant parameters to be consider for hypoxia induction in in vitro cell cultures.

Parameter	Effects on O_2_ Delivery	References
Cell type	Cells from different tissues have different O_2_ consumption rate (OCR) that influences O_2_ delivery.	[63]
Culture geometry dishes	O_2_ diffusion through polystyrene changes between culture dishes geometries and it is responsible for up to 30% of O_2_ delivery.	[58]
Seeding density	Seeding density influences the OCR. Experiments should be performed using always same cell densities, avoiding confluent cell cultures.	[59]
Medium volume	Culture medium holds less O_2_ than air per unit volume and limits the movement of O_2_ molecules.	[59]
	Medium depth determines the diffusive barrier to O_2_ delivery.	[58]
Medium composition	Protein and glucose, normally added to culture medium, reduce the capacity for dissolved O_2_	[59]
Temperature	Cold medium holds significantly more O_2_ than warm one.	[58]
	Increases in temperature cause conflicting effects of increasing the diffusion coefficient while decreasing O_2_ solubility. As medium is cooled, the O_2_ solubility increases.	[58]
	The concentration of dissolved O_2_ depends on the temperature and partial pressure of O_2_ in the gaseous phase.	[59]
Humidity	Humidity with carbon dioxide dilutes other atmospheric components–for dry air moving to saturation (~6% water vapor) and 5% CO_2_ reduces the partial pressure of O_2_ by 11% (or 8% for an initial atmosphere at 50% relative humidity).	[58,59]
Altitude	The decrease of atmospheric pressure with an increasing altitude, influences the amount of O_2_ in the cell culture medium.	[52]
Handling cell cultures	Moving cells from hypoxia to ambient air generates O_2_ fluctuations that influences O_2_ concentration.	[60]

Another approach to reproduce hypoxia in cell culture is represented by pharmacological treatment with chemical agents defined as “hypoxic mimetic compounds” (detailed in Section 3.3), among which cobalt chloride (CoCl_2_) is one of the most used [64]. The chemical induction is cheap, easy to perform in cell cultures, allowing operators to open culture dishes or flasks without affecting hypoxic conditioning. However, these compounds may possess, in addition to HIF-1α induction [65], other unknown effects that limit the use depending on cells type and density and it is necessary to test preliminary their potential toxic effects to define the best concentration for inducing hypoxia without affecting cell viability. The time of exposure to hypoxia is another parameter to be considered as cells differently tune gene expression depending on short-or long-term hypoxia exposure [57,66]. Hypoxic induction with chemical compounds is kept just for a short period in cell cultures (maximum of 72 h usually), while performing physical hypoxia allows to maintain cultures in low O_2_ condition for long term [64]. Moreover, keeping low O_2_ conditions for a long time of exposure, allows cells to adapt to hypoxic environments justifying the variety of cellular outcomes. On the other hand, different cellular responses to hypoxia can be also dependent on different HIFs isoforms activated since they have a specific temporal role within cells [67]. Even if both HIF-1 and HIF-2 isoforms mediate the hypoxic response overlapping and target genes, it has been demonstrated that HIF-1 drives the early response to hypoxia within 24 h, while HIF-2 seems to manage the chronic response after 24 h, creating the so-called “HIF switch” mechanism in cells [67].

Literature data suggest that O_2_ levels in standard cell culture experiments significantly deviate from a physiological range, as well as show that O_2_ levels vary dramatically under different experimental settings, cell types investigated, cell confluency, volume and timing of media exchange, etc. (Table 1 and Table 2).

In the following paragraphs some and most used approaches for in vitro hypoxia induction in cells culture are detailed.

### 3.1. Methods Providing Physical Hypoxia Conditions: Hypoxic Chambers, Tri-Gas Incubator, and Hypoxic Workstation

**Figure 5:**
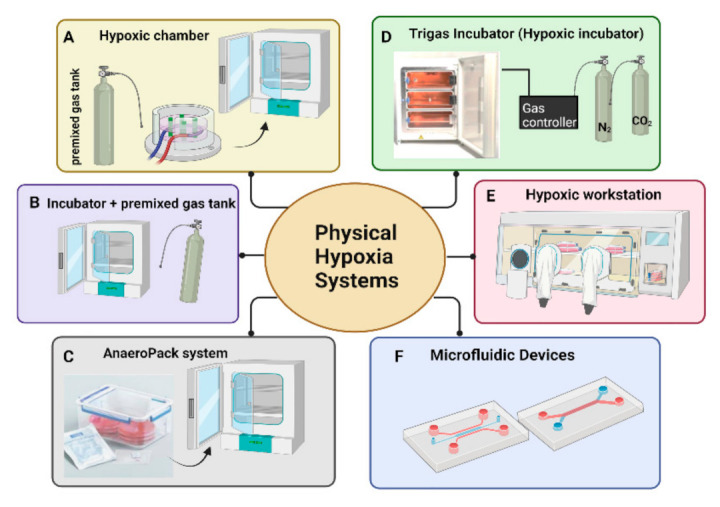
Most relevant Systems used to induce in vitro physical hypoxia. Gas mixture from a single tank can be connected directly to (**A**) hypoxic chambers before their incubation in standard CO_2_ incubator or (**B**) incubator. A similar mechanism is used for (**C**) AnaeroPack system. (**D**) “Tri-Gas” incubator: only two gases are supplied, CO_2_, as usual, and N_2_, that can be modulated to reduce O_2_. (**E**) hypoxic workstation. (**F**) example of microfluidic devices.

Different systems to reproduce low O_2_ levels for in vitro cell culture has been proposed in the literature (Figure 5). Incubators or hypoxic chambers are the most used systems (Figure 5A,B). The easier way is represented by the use of modular gas chambers inside a standard CO_2_ incubator. For investigators who want to test hypoxia effects for their own cells and projects, these small chambers could be a good solution. The chamber is made of solid materials in a fixed shape and size holding up to twelve 10-cm dishes and require additional equipment, as regulators, tubing, and pumps for gassing the chamber with a pre-mixed gas mixture. They must be recharged after each entry, and currently cannot be monitored for internal conditions. The inclusion of an extra dish with sterile water maintains the humidity within the chamber [68]. Hypoxic chambers were mostly used in the past decades. However, one of the common defects of this chamber is leakage, although it is not frequent, and the generation of an inner pressure if the operation is inappropriate. For this reason, most hypoxic cultures today are performed in a “Tri-gas” incubator (Figure 5C), a not properly adequate definition because only two gases CO_2_ and N_2_ are supplied causing the reduction of O_2_ that can be lowered to 0.5–1%. Some manufacturers may claim O_2_ levels as low as 0.1%, but this is hard to achieve due to the sensor detection limits. The first commercially available “Tri-gas” incubator was released in 1979 [68]. Inside the incubator can be placed separated compartments with their own glass doors reducing the O_2_ fluctuations but also contaminations. The use of this incubator proved that cells in hypoxia grew better, healthier, and with longer lifespans. Other incubators reproduce hypoxic conditions using a gas mixture from a single tank without separate sensors, but this prevents cells from receiving proper amounts of premixed gases [68].

Even if “Tri-gas” incubators can provide a hypoxic environment, they do not protect cells from ambient conditions during any procedures that must take place outside the incubator, such as medium changes. This extra exposure to higher levels of O_2_ concentration negatively impacts cell growth. It has been reported that few minutes of exposure to ambient O_2_ conditions accumulated over several months adversely affect the results of culturing experiments [58]. Like the hypoxic chambers are the anaerobic bags (AnaeroPack) (Figure 5D), an innovative system that we found in two articles of bibliometric research [69,70]. These bags are very easy to handle without requiring water or catalyst, simply putting AnaeroPack in jars. This system can reproduce a suitable atmosphere specially to grow microorganisms allowing three types of cultivation (i.e., anaerobic, microaerophilic, and CO_2_ cultivation with selected concentrations). The major advantages of this system are their low cost and easy handling, without preparing large equipment and generating high temperature. Furthermore, the use of a hypoxia workstation [60], which can offer precise control of O_2,_ and CO_2_, seems to be an appropriate method (Figure 5E). The hypoxic workstation keeps cells to constant O_2_ levels because cells can be passaged, and culture media can be changed without altering O_2_ levels within it; cells can be handled for experiments or lysates preparation preventing all those O_2_ dependent modifications that may alter some cellular constituents [60]. These operative functions are possible because the workstation has a chamber equipped with O_2_ sensors to monitor the O_2_ concentration and two gloves access ports for sample handling [60]. The workstation is especially useful for those researchers that need almost an “anoxic” environment or with very low O_2_ concentrations difficult to manage with the use of a traditional incubator. However, “Tri-gas” incubators and hypoxic workstations are expensive, and this makes them not convenient for small laboratories that do not perform hypoxia experiments routinely.

The CulturePal (Trial Products; Mitsubishi Gas Chemical Company Inc.) with built-in deoxidizing reagent [71], represents a novel system for the induction of a hypoxic atmosphere. The principal constituent is sodium ascorbate, which absorbs O_2_ and generates CO_2_ by oxidative degradation [71]. In their experiment, Ito and colleagues [71] adopted two series of this system: the CulturePal-Zero, for the modulation of O_2_ levels <0.1%, and CulturePal-Five (3–7% O_2_ levels). According to the authors, CulturePal systems would be a suitable system for the induction of short-term hypoxia and the regulation of gas concentrations during cell transportation [71].

Recently, a sophisticated Microfluidic Devices have been proposed to reproduce a hypoxic environment with a precise control of O_2_ tension over temporal dimension and spatial one, in order of microns (Figure 5F). The small dimension of this device allows to minimize the distance of O_2_ diffusion creating a microvascular system of small volumes (in the range of microliters) [72]. An example of these microfluidic systems is represented by laser-cut polycarbonate foils, produced with a layer-by-layer manufacturing technology, and an elastomeric membrane joined together using thermal diffusion bonding. Mechanical strength, chemical resistance, and biocompatibility characterized the fluidic layers. Several O_2_ sensing spots are integrated into the device and monitored O_2_ content helping to adjust its levels and thus producing stable and defined hypoxic conditions for cells [73]. Another chip, described by Barmaki et al., utilizes two separate, but interdigitated microfluidic channels. The hypoxic microenvironment was created by sodium sulfite as an O_2_ scavenger in one of the channels and started to increase after 100 min of pumping in the single channel [74]. Mathematical simulations contribute to support O_2_ diffusion measurements rendering this kind of system very accurate [75].

### 3.2. Biological-Mediated Approaches of Hypoxia: Spheroids 3D Cultures

Tissues are characterized by the presence of a gradient of O_2_, nutrients, and paracrine factors but this state is not easily reproducible in typical 2D cultures [76]. For this reason, 3D culture model has received increased scientific interest as a favorable condition because it is closer to the physiological native environment [77] (Table 2).

The 3D culture is a common term used to refer to spheroid cultures, a method largely adopted for cancer cells cultures, but recently also to reproduce the physiological hypoxia cell niches [78]. Indeed, 3D in vitro model approaches may reproduce hypoxic conditions (Table 2) because of the lower O_2_ concentration in the inner part of spheroids [79]. Although this is not properly a method to induce in vitro hypoxia, it has been hypothesized that spheroid formation potentiates cell function by the generation of a hypoxic core within spheroids with sufficient large diameters [79]. The spheroid size is a considerable variant as the difficulty in controlling spheroid diameters has an impact on the diffusion of nutrients, signaling molecules and O_2_ concentration which decreases near the spheroid core [80].

Different developed methods, such as hanging drop, chitosan film cultures, or the use of bioreactors and rotating cultures [81] provide a suspension culture condition in which cell–cell adhesion and cell–matrix interactions improve the self-assembly of cells leading to the spheroid formation or 3D tissue-like aggregates [76].

Under these culture conditions, cells are stimulated to grow with the formation of numerous 3D proliferation centers, hypoxic regions, and specific microenvironments that allow them to express a tissue-like phenotype. The tendency of cells to form spheroids could be interpreted as a marker of the undifferentiated state of cells as stem cells. Indeed, literature data evidenced that adult MSCs possess a remarkable ability to coalesce and assemble in tri-dimensional (3D) structure which closely recapitulates the in vivo MSCs niche. MSCs cultured in 3D spheroid cultures showed a stable immuno-phenotypic profile, with a significant enhancement in survival, homing, stemness features differentiation potential, angiogenic effect, and anti-inflammatory properties [82]. The most relevant effects on 3D spheroids cultures are the high expansion and colony formation, the differentiation potential, and epigenetic changes in pluripotent genes such as *Oct-4*, *Sox-2*, and *Nanog* [83].

Zhang et colleagues observed that gingiva mesenchymal stem cells (GMSCs) spheroid expressed higher levels of HIF-1α and HIF-2α, against the adherent counterparts and increased production of ROS thus recapitulating features of low O_2_ conditions [82]. Even if the correlation between hypoxic core within spheroids and HIF activation is well described in oncological research (solid tumors are characterized by regions permanently or transiently hypoxic due to the poor blood supply and lack of vascularization), the hypoxic adaptations in spheroids rely on the activation of the transcription factor HIF [84] and its influence on the maintenance of multipotency and self-renewal [55]. Consistent with this hypothesis, hypoxia-regulated genes, such as *VEGF*, are upregulated in MSCS spheroids [85].

An additional aspect to be considered in managing 3D cell cultures is the O_2_ gradient as the three-dimensionality and the variable thickness of cellular structures introduce additional irregularities that hamper oxygen diffusion and lead to the formation of O_2_ concentration gradients. In that context, several novel approaches and techniques have emerged tackling the challenges of O_2_ gradient concentration as the use of oxygen-sensing microelectrodes [61]. However, the invasive nature of the approach represents a disadvantage as since time and technical challenges require repetitive calibrations and measurements in different spots inside the tissue construct motivating the search for alternative approaches.

For this aspect, the use of 3D cell culture systems for in vitro hypoxia induction is represented by the possibility of actively inducing a controlled O_2_ gradient across the model, based on the experimental needs. These gradients can be induced with different methods, by perfusion with an oxygen scavenger in the medium, by positioning the culture between two micro-channel circuits perfused with gas, or by incorporation of an O_2_-consuming reaction of specific hydrogel and thus regulating the O_2_ levels [52].

### 3.3. Hypoxia Mimicking Agents

Some in vitro models utilize “hypoxia mimetic agents” biological or chemical molecules which simulate hypoxic conditions predominantly by increasing the availability of intrinsic HIF-1α in standard cell culture settings. This methodology can be used for both sustained and intermittent hypoxia models, the latter of which can be achieved by cyclic exposure to the agent. The precise mechanism of action of hypoxia mimetic agents may vary depending on the particular agent used (Table 2). Transcriptionally active HIF levels rise at sub-physiological concentrations of O_2_ inducing upregulation of a range of genes with activities ranging from cell protection to apoptosis according to the specific context and cell type. As reported in Section 1.3, the regulation of HIF degradation requires hydroxylation by PHDs [56]. The PHDs are 2-oxoglutarate (2OG) and non-heme-Fe (II)-dependent dioxygenase family members, all requiring ferrous iron (Fe^2+^), 2OG, O_2_, and ascorbate for the enzymatic activity. Indeed, the reduction of substrate hydroxylation results in HIF-1α stabilization [86]. However, since HIF-1α is not the exclusive substrate of PHDs, it must be taken into account that its stabilization via PHDs inhibition could affect also other pathways [56].

The most used HIF stabilizers “hypoxia mimetic agent” are CoCl_2_, dimethyloxalylglycine (DMOG), and deferoxamine (DFO) which hamper HIF degradation by the inhibition of PHDs although with different mechanisms [87].

More in detail, DMOG is a competitive inhibitor of the three PHD isoforms and of factor inhibiting HIF (FIH). DMOG acts as an analog of 2OG (co-substrate of PHDs), placed at the catalytic site-blocking enzymatic activity [87].

DFO is a Fe^2+^ chelator, another essential cofactor in PHD activity. A lack of Fe^2+^ availability causes inhibition of the activity of PHDs and FIH, provoking HIF-1α accumulation and an increase in activity [88].

The hypoxic CoCl_2_ model is based on the inhibition of PHDs by substitution of the Fe^2+^, thus increasing HIF-1α protein levels and inducing its transcriptional activity. However, experimental results have suggested different speculations regarding the mechanism of HIF-1α stabilization by Co^2+^ [64]. It has been shown that cobalt can prevent the binding of HIF-1α to von-Hippel–Lindau protein (pVHL), block HIF-1α degradation, or deplete ascorbate which is essential for maintaining the PHDs in the active state. An increase in HIF-1α levels after CoCl_2_ treatment could be linked also to ROS generation [89].

However, even if the effects of hypoxia-mimetic agents are comparable to those resulting from reduced atmospheric O_2_ levels [90], it should be noted that one of the most common downsides is their potential cellular cytotoxicity [52]. So, a hypoxic environment is induced by stabilization and accumulation of HIF-1α that occur because all of these chemical agents block the activity of the PHDs, disrupting the hydroxylation of HIF-1α and inhibiting the ubiquitin-dependent 26S proteasomal degradation pathway. In addition, they also have inhibitory effects on FIH. In this context, a newly developed PHD inhibitor, namely JNJ-42041935 1-(5-Chloro-6-(trifluoromethoxy)-1*H*-benzimidazol-2)-1*H*-pyrazole-4-carboxylic Acid (JNJ) has been identified through structure-based drug design methods and it seems to be highly selective for all the isoforms of PHDs relative to FIH [91]. Moreover, JNJ showed a high efficiency in stabilizing both HIF-1α and HIF-2α isoforms [92].

#### Other Hypoxic Mimetic Agents

Other stimuli and unusual chemical compounds are often used to activate HIF-1α and to mimic hypoxia (Table 2). Among these, there is ferulic acid (FA), a phytochemical found in the walls of plant cells with potential therapeutic effects in wound healing and ischemic diseases. It has been shown that FA can upregulate HIF-1α, VEGF, and platelet-derived growth factor (PDGF), which subsequently activate mitogen-activated protein kinase (MAPK) and phosphatidyl inositol 3-kinase (PI3K) pathways, improving angiogenesis [93].

Qiu et colleagues investigated the beneficial effect of FA on stemness of human tendon-derived stem cells (hTSCs) and demonstrated that FA treatment promoted proliferation, self-renewal, and multi-differentiation potential (adipogenesis, chondrogenesis, and osteogenesis) in hTSCs cultures, in a dose-dependent manner. However, the authors suggest that, in in vitro experiments, high FA concentration might present slightly adverse effects on cells suggesting that FA beneficial action falls within an optimal range [94]. Then, the critical involvement of HIF-1α in mediating the FA-elicited pro-stemness effect on hTSCs was further disclosed by a specific knockdown assay, which readily abolished this beneficial influence of FA [94].

Another natural product is celastrol, extracted from the Tripterygium wilfordii Hook, which, together with a variety of biological effects [95], appears able to stimulate hypoxia by HIF-1α stabilization protein synthesis [96]. It has been demonstrated that in hTSCs the celastrol treatment induced in vitro hypoxia via HIF-1α accumulation and significantly enhanced stemness of hTSCs in a HIF-1α dependent manner. Specific knockdown assay confirmed not only the function of HIF-1α in mediating celastrol pro-stemness effect on hTSCs, but also identified the mechanism of celastrol action in the HIF-1α-Smad7 axis pathway [96].

Additionally, an environmental factor such as ultraviolet A irradiation (UVA) is considered as hypoxia inductors on cells since they modulate HIF-1 expression. Indeed, the HIF-1 pathway seems to be susceptible to UVA which exerts an adverse effect on cells by promoting senescence [97], reducing the expression of stemness genes by activation of Prostaglandin E2 (PGE2)-cAMP-HIF-1α signaling [98]. UVA reduces the expression of stemness genes such as *Oct-4*, *Nanog*, and *Sox-2* through the downregulation of HIF-1α. Using an HRE-luciferase reporter assay it has been shown that the UVA irradiation reduced mRNA level of HIF-1α, negatively modulating stemness genes [99]. With a screening tool, several molecules have been selected as ideal candidates for reverting the negative UVA effect on stemness, by recovering HIF-1α stabilization via the inhibition of the PGE2-cAMP signaling in hMSCs from adipose tissue among which there are sinapic acid [100], aspartic acid [99], arctigenin [101], and ethylcystein [98].

Most interestingly, some other factors that are commonly used in cell cultures, such as glucose, normally added to the culture medium, can modulate HIF-1α protein expression by reducing the capacity to dissolved O_2_ [59].

Several studies demonstrated that high-glucose may influence HIF-1α expression in various mammalian cells. As reported in literature, high glucose concentrations can increase intracellular superoxide levels leading to reduction of HIF-1α expression [102].

In nucleus pulpous-derived mesenchymal stem cell (NPMSC), cultured in high glucose condition, a lower expression of HIF-1α has been detected compared with NPMSC cultured with low glucose. At the same time, high glucose concentrations induce senescence and significantly decrease proliferation and stemness maintenance as indicated by the reduction of stemness genes expression (*Sox-2*, *Nanog*, and *Oct-4*), and this effect could be linked to HIF-1α reduction [103].

### 3.4. In Vitro Induced Hypoxia Using Multifactorial Approaches

Considering the difficulty of keeping a constant level of O_2_, especially working within hypoxic chambers, it could be useful to add chemical compounds in a short range of time to preserve the stabilization of HIF protein. In this context, Večeřa and colleagues, induced hypoxia in NSCs cultivating them in an anaerobic chamber with 1% of O_2_ and contextually treating cells with 300 µM of CoCl_2_ for 6 h. Under this condition the NSCs were able to display their stemness features [104]. It has been shown that CoCl_2_ can induce stemness maintenance in MSCs promoting the expression of stemness markers as Oct-4, Sox-2, or Nanog [105]. On the other hand, CoCl_2_ can limit MSCs expansion inducing significant apoptosis due to the loss of the downstream nuclear factor of erythroid-derived 2-like 2 NRF2 [106]. In this context, the combinatorial overexpression of NRF2 and treatment with CoCl_2_ could restore the maintenance of MSCs characteristics, promoted by CoCl_2_ treatment, preventing apoptosis [106]. Similarly, in BMSCs, IGF-1 overexpression could restore Oct-4 and Nanog expression that decreased under 1% O_2_ condition, performed with AnaeroPack system [69].

The possibility of combinate low O_2_ culture with other culture conditions represents a system for the improvement of cellular response. For example, beneficial effects of hypoxic exposure combined with treatment with 1.8 mM of calcium ions (Ca^2+^) have been reported on proliferation and self-renewal ability of small MSCs that showed also higher resistance response to passage-dependent senescence [107]. In the same way, in hUCB-MSCs treatment with hypoxia and Ca^2+^ exposure increased proliferation without losing Oct-4 and Nanog stem cell markers expression that resulted significantly higher in comparison to same cells treated with Ca^2+^ or 3% O_2_ concentration alone. This combinatory approach was able to also enhance the hUCB-MSCs differentiation potential suggesting that the synergistic effect of Ca^2+^ and hypoxia in stem cells was dependent on HIF-1α expression and its downstream extracellular signal-regulated kinase (ERK) pathway [108].

Hypoxic culture can be easily coupled with seeding on chitosan films, as reported for cultured equine umbilical cord mesenchymal stem cells (UCM-MSCs), which showed an increase of *Oct-4*, *Sox-2*, and *Nanog* genes expression, after 7 days of culture [109]. These synergistic effects strongly support that the hypoxia and factor combinatory approach could be considered a good strategy to enhance the stemness potential of these cells to improve their positive response in healing tissues [110].

In a recent study performed in primary human Wharton’s jelly MSCs, hypoxia has been induced by a combination of 5% O_2_ levels with a pressure stimulus 2.0 PSI by using an AVATAR system increase of proliferation rate of cultured cells [111].

However, it is considered that not always the combination of more factors results in enhanced positive effects for cells. Indeed, even if hMSCs 3D spheroids and low O_2_ culture were able to enhance stemness gene expression when used in e separate manner compared to flat substrate culture, their combination was not able to increase hMSCs stemness markers expression while maintaining Oct-4, Rex-1, and Sox-2 at constant levels also in spheroids exposed to different O_2_ concentration [112].

**Table 2:** Summarized scientometric research articles for hypoxia induction and its effect on stemness modulation, in in vitro cell cultures.

Cell Source	Chemical Induction	Time of Exposure	HIF Analysis	Hypoxic Effect on Stemness	Reference
rat BM-MSCs (bone marrow derived MSCs)	BMC-CM (bone marrow cells conditioned medium)	1,3,5 passages	RNA expression (*HIF-1α*)	BMC-CM increases HIF-1α which suppresses OXPHOS activity and activates the anaerobic glycolytic metabolic pathway.	[113]
rat NPMSCs (Nucleus Pulposus mesenchymal stem cells)	High Glucose 4, 5 g/L vs. Low Glucose 1 g/L	3 passages	Protein expression (HIF-1α)	A significantly decreased expression of HIF-1α, Oct-4, Sox-2, Nanog, and GLUT-1 were found in High glucose NPMSCs in comparison to low glucose NPMSCs.	[103]
human TSCs (tendon stem cells)	Ferulic Acid (2, 10, 15 µM)	48 h	RNA, protein expression ChIP assay (HIF-1α)	Increase of self-renewal ability: colony number and average colony size were markedly increased in response to FA treatment.	[94]
human TSCs (tendon stem cells)	Celastrol (1, 2, 4 µM)	24 h	RNA, protein expression, ChIP assay (HIF-1α)	Improved self-renewal capacity evaluated through the proliferation rate and colony formation assays.	[96]
human SHED (Stem cells from human exfoliated deciduous teeth)	CoCl_2_ (50, 100 µM)	3 days	Protein expression (HIF-1α)	Increase of stemness markers expression (Oct-4, Sox-2, Nanog, and c-Myc).	[114]
human DPSCs (Dental pulp stem cells)	CoCl_2_ (10 µM)	48 h	RNA expression (*HIF-1α*)	Increase of stemness markers expression. (Oct-4 and Sox-2)	[115]
human UC-MSC (Umbilical cord derived mesenchymal stem cells)	CoCl_2_ (5, 10 µM)	12, 24, 72 h	Protein expression and immuno-detection	Increase Nanog and NRF2 (nuclear factor erythroid-derived 2-like 2) expression. However, CoCl_2_ limited MSCs expansion as it induced significant apoptosis that can be recovered with NRF2 overexpression.	[106]
human DPSCs (Dental Pulp Stem cells)	CoCl_2_ (25 µM, 50 µM)	3 d	n.d. (Treatment with Apigenin, an HIF inhibitor, reverts CoCl_2_ effects)	Increase of stemness markers expression, significant with 50 µM (Rex-1, Oct-4, Sox-2, and Nanog).	[105]
human AAA-MSCs (Mesenchymal stem cells from abdominal aortic aneurism)	CoCl_2_	24 h, 48 h, 72 h	n.d.	No differences in stemness gene expression. Stemness profile is unaffected by hypoxic treatment.	[116]
human PDLSCs (Periodontal ligament stem cells)	CoCl_2_ (50 µM, 100 µM)	1, 3, 7 d	Protein expression (HIF-1α and HIF-2α)	Increase of stemness markers expression (7dRex-1 and Oct-4).	[117]
human ADSC (Adipose derived mesenchymal stem cells)	Arctigenin (1, 10, 50 µM)	3 d	RNA and protein expression	Increase stemness markers expression by antagonizing UVA irradiation effect. The effects of arctigenin are mediated by PGE2-cAMP signaling-dependent upregulation of HIF-1α.	[101]
human AMSCs (Adipose tissue derived mesenchymal stem cells)	Sinapic Acid (20, 200, 400 µM)	3 d	RNA and protein expression	Increase stemness markers expression by antagonizing UVA irradiation effect. The effects of sinapic acid are mediated by PGE2-cAMP signaling-dependent upregulation of HIF-1α.	[100]
human AMSCs (Adipose derived mesenchymal stem cells)	Aspartic Acid (1, 10, 100 µM)	3 d	RNA and protein expression	UVA irradiation decreases stemness via HIF-1α downregulation. Aspartic Acid increases stemness marker via upregulating HIF (antagonizing UVA irradiation).	[99]
human ASCs (Adipose derived mesenchymal stem cells)	Ethylcystein (1, 10, 200 µM)	3 d	RNA and protein expression	UVA irradiation decreases stemness via HIF-1α downregulation. Ethylcysteine recovers stemness by increasing HIF-1α levels.	[98]
**Cell Source**	**3D Cultures**	**Time of** **Exposure**	**HIF Analysis**	**Hypoxic Effect on Stemness**	**Reference**
Human MSCs, Human TMSC (Turbinate Mesenchymal Stem Cells) human ADSC (adipose derived stem cells)	3D cultures	7 d	n.d.	stemness is related to spheroids size. ADSCs expressed stemness markers Oct-4 and Nanog.	[118]
human DPCs (Dental pulp cells)	3D cultures	1, 4, 15 d	n.d.	compared to monolayer DPCs, spheroids showed higher expression levels of stem cell markers, Nanog, CD44, and TP63.	[119]
mouse GMSCs (Gingiva derived mesenchymal stem cells)	3D cultures	Up 3 d	Immunodetection (HIF-1α and HIF-2α)	Increase of stemness markers expression Oct-4 and Nanog.	[82]
human UCMSCs (umbilical cord derived mesenchymal stem cells)	Hypoxic chamber (n.d.)/3D culture	2 days	Protein expression	Maintenance of stemness is related to the 3D cultures. Hypoxia is used to test resistance to hypoxic stress.	[77]
**Cell Source**	**Combinatorial Methods**	**Time of** **Exposure**	**HIF Analysis**	**Hypoxic Effect on Stemness**	**Reference**
mouse NECs (neuroepithelial cells)	Hypoxic chamber 1% O_2_ + CoCl_2_ 300µM	13 d + 6 h CoCl_2_	Protein expression + co-IP and ChiP (HIF-1α)	Preservation of neural stemness via *Hes1* (hairy enhancer of split 1). HIF deficient-neurospheres showed reduced self-renewal properties and decreased expression of Tbr2, a marker of proliferating basal progenitors.	[104]
human UCB-MSC (umbilical cord blood MSCs)	3% O_2_ + 1.8 mM Calcium	5 days	n.d.	Increase of stemness markers expression related to primitive stem cells including Oct-4, Nanog, STELLA, SALL-4, and BMI-1.	[107]
horse UCM-MSCs (umbilical cord matrix derived mesenchymal stem cells)	Incubator 5% O_2_ + seeding on chitosan films	3 d and 7 d	n.d.	Increase of spheroids formation and size; increase of stemness markers expression, Oct-4, Sox-2, and Nanog.	[110]
horse UCMSCs (Umbilical cord derived mesenchymal stem cells)	Incubator 5% O_2_ + seeding on chitosan films	3 d and 7 d	n.d.	7 d Hypoxic cultures of + seeding on chitosan films increases stemness	[109]
human PMSCs (Placenta derived stem cells)	Incubator 5% O_2_ + HIF2α over-expression	4 h/24 h	Protein expression (HIF-2α)	Increase of stemness markers expression, CCND1 (CyclinD1), c-Myc, and POU5F1 (Oct4).	[120]
human MSCs (Mesenchymal stem cells)	Incubator 2% O_2_ /3D cultures	From 24 h to 96 h	n.d.	Oct-4, Rex-1, Sox-2, and Notch-1 levels did not change significantly in spheroids, between different O_2_ culture conditions. However, compared to flat substrate culture, *Sox-2* and *Notch-1* gene expression was significantly increased in low O_2_ spheroids.	[112]
human ATSCs (Adipose tissue stromal cells)	Hypoxic chamber 1% O_2_ + 10 ng/mL DHP (4-3,4-dihydroxyl phenyl)	2–6 h	Protein expression (HIF-1α)	Hypoxia + DHP increases stemness markers by inducing de-differentiation on ATSCs. De-differentiated ATSCs overexpress Oct-4, Sox-2, Nanog, Rex-1, and c-Myc.	[121]
human WJMSCs (Wharton’s jelly MSCs)	Incubator 5% O_2_ + pressure stimulus 2.0 PSI	24, 48, 72 h	n.d.	Increase of proliferation rate under hypoxic condition + pressure stimulus.	[111]
**Cell Source**	**Physical Hypoxia**	**Time of** **Exposure**	**HIF Analysis**	**Hypoxic Effect on Stemness**	**Reference**
human MSCs (Mesenchymal stem cells)	Culture Pal System <0.1% and 5% O_2_	24 h, 72 h	n.d.	No stemness markers but low O_2_ suppresses cell senescence through down-regulation of p16^INK4A^ mRNA expression.	[71]
mouse TSCs (Trophoblast stem cells)	Anaerobic bags 0% O_2_; Incubator 0.5% and 2% O_2_	12 h	n.d.	0.5% O_2 ×_ 12 h causes loss of ERRB2 and ID2 (specific stemness markers); while 2% O_2_ × 12 h maintains potency.	[70]
mouse TSCs (Trophoblast stem cells)	Hypoxic chamber 0.5% O_2_	from 1 to 6 d	n.d.	0.5% O_2_ reduces stemness in favor of differentiation.	[122]
rat BMSCs (Bone marrow stem cells)	AnaeroPack system in anaerobic jar 1% O_2_	48 h	n.d.	Hypoxia decreases stemness. Overexpression of IGF1 maintains stemness under hypoxia.	[69]
human HSPCs (hematopoietic stem/progenitor cells) coculture with WJMSC	Incubator 1% O_2_	7 days	RNA expression (*HIF-1α—HIF-2α*)	Hypoxia activates the Notch/Wnt/Hedgehog signaling pathway which plays an important role in preserving stemness.	[123]
human ESCs (Embryonic stem cells)	Incubator 1% O_2_	-	HIF-1α is one of the top HUB gene	Hypoxia activates pathway involved in stemness maintenance.	[124]
rat MMSCs (Metanephric Mesenchymal stem cells)	Incubator 1% O_2_	3 d	Protein expression (HIF-1α)	Decrease of stemness markers (Six-2/Cited-1 are specific markers of MMSCs).	[125]
human BMMSCs (Bone Marrow Mesenchymal stem cells)	Hypoxic chamber 1% O_2_	1–2 weeks	n.d.	Increase of Oct-4 expression.	[126]
mouse BMMSCs (Bone Marrow Mesenchymal stem cells)	Incubator 1% O_2_	6 weeks	RNA expression (*HIF-1α—HIF-2α*)	Increase of c-myc expression and colony numbers.	[127]
human ob/nV-ASCs (Adipose stem cells from Visceral fat of obese individuals/non obese)	Hypoxic chamber 1% O_2_	2, 4, 8 h	RNA and protein expression (HIF-1α)	obV-ASCc obV-ASC, which showed a less stem-like phenotype, recovered stemness features after hypoxia. Increase of KLF4 and Oct-4 expression (after 8 h of hypoxia).	[128]
human MSCs (Mesenchymal stem cells)	Incubator 1% O_2_	7 d	n.d.	Increase of stemness markers expression, Oct-4, Klf4, and c-myc.	[129]
human BMMSCs (Bone Marrow Mesenchymal stem cells)	Incubator 1% O_2_	1, 3, 5, 7 d	n.d.	Increase of stemness markers expression (7 d) Oct-4, Nanog, Klf4, and Sall4. Hypoxia increases proliferation and cyclin D1 (CCD1) expression.	[130]
human ASCs (Adipose derived mesenchymal stem cells)	Incubator + hypoxic workstation 1% O_2_	21 d	n.d.	Expansion of ASCs in 20% O_2_ led to a significant decrease in T/S ratio (relative length of the telomeres) compared to 1% O_2_. ASCs in 1% O_2_ proliferates faster, shows reduced aging, and preserves stemness.	[131]
mouse SPCs (Cochlear stem progenitor cells)	Incubator 1% O_2_	24 h	RNA and protein expression (HIF-1α)	Increase of stemness markers expression, Nanog, Oct-4, and Musashi1.	[132]
human BMMSCs (Bone Marrow Mesenchymal stem cells)	Hypoxic chamber 1% O_2_	2 passages /Until senescence	n.d.	Increase of stemness markers expression, Nanog, Oct-3/4, and Sox-2.	[133]
mouse ESCs (Embryonic stem cells)	Hypoxic chamber 1% O_2_	24 h	RNA expression (*HIF-1α*)	Hypoxia favors differentiation through *H2afz* gene downregulation. While *H2afz* overexpression maintains stem markers (Nanog, Rex-1, andFgf4)	[134]
human ADMSCs (Adipose derived mesenchymal stem cells)	Hypoxia Incubator chamber 1–3% O_2_	7 d	n.d.	Nanog and Sox-2 increased under low O_2_ tension, although the differences were not statistically significant.	[135]
Mouse ESCs (Embryonic stem cells)	Incubator 1% O_2_ or 5% O_2_	24 h, 48 h	RNA and Protein expression (HIF1-α and HIF-2α)	Reduction of stemness.	[92]
human MSC in co-culture with HUVEC (human umbilical vein endothelial cells)	Incubator 2% O_2_	7 d	RNA expression (*HIF-1α*)	Expression of stemness genes was lowered due to adipogenic differentiation of MSCs.	[136]
human ASCs (Adipose derived stem cells)—fresh vs. cryopreserved cultures	Incubator 2% O_2_	3 passages	RNA expression (*HIF-1α*)	Increase stemness markers (Nanog, Sox-2, Oct-4, and Rex-1) especially in fresh hypoxic cultures vs. fresh normoxic ones.	[137]
human ASCs (Adipose derived mesenchymal stem cells)	Incubator 2% O_2_	3 passages?	RNA expression (*HIF-1α*)	Increase of proliferation and stemness markers expression, Rex-1, Oct, Sox-2, and Nanog).	[138]
human PDLSCs (Periodontal ligament stem cells), DPCs (Dental pulp cells)	Hypoxic chamber 2% O_2_	24 h–1 w	Protein expression (HIF-1α)	Increase of stemness markers expression (Oct-4, Sox-2, and c-Myc) and 3D niche-like structures.	[139]
human HUCPVCs (Umbilical cord perivascular cells)	Incubator 2% O_2_	2 w	Protein expression (HIF-1α and HIF-2α)	Increase of Oct-4 expression and colonies number.	[140]
mouse ASCs (Adipose derived mesenchymal stem cells)	Incubator 2% O_2_ (100 mM CoCl_2_ used aspositive control for HIF)	Passages 1, 5, 8over 6 w	Protein expression (HIF-1α)	Increase of stemness markers expression, Oct-3/4 and Nanog, at passage 5.	[141]
human MSCs (Mesenchymal stem cells)	Incubator 2% O_2_	52–64 d	n.d.	Hypoxia prevents senescence since hypoxic hMSCs maintained their homogenous rapidly self-renewing morphology for up to 52 days.	[142]
rat CDCs (Cardiosphere derived cells)	Incubator 2% O_2_or DMOG 1 mM or BIC 30 µM	P2 until 80% confluency	RNA and protein expression (HIF-1α)	Increase of stemness markers expression: Oct-4, Sox-2, Klf-4, Nanog, and c-Kit. Increase of GLUT-1 expression, lactate production, and glucose uptake	[143]
human UC-MSC (Umbilical cord derived mesenchymal stem cells)	Incubator 2.5% O_2_	15′ 2,5% O_2_ + 30′ 21% O_2_ + 72 h 2.5% O_2_	n.d.	Absence of stemness markers both in hypoxic and normoxic group: no expression of *Oct-4*, *Nanog*, and *Nt-3* genes were detected.	[144]
human ASCs (Adipose derived mesenchymal stem cells)	Incubator chamber 2–3% O_2_	6 d	n.d.	Increase of stemness markers expression, Nanog and Sox-2.	[145]
human WJ-MSCs (Wharton’s jelly mesenchymal stem cells)	Hypoxic chamber 3% O_2_	3–6 days	RNA expression (*HIF-1α*)	Increase of stemness markers expression, of Nanog, Oct-4A, Oct-4B, and Sox-2. Enhanced WJ-MSCs clonogenicity and expansion capacity.	[146]
human DPCs (Dental Pulp Stem cells)	Hypoxic chamber 3% O_2_	2 days	Protein expression (HIF-1α)	The expression of the cell surface markers, CD133, CD34 is increased, while CD105 and Oct-4 do not change significantly. Increase of colony forming units. ROS reduction under hypoxia.	[147]
human CDCs (Cardiac stem cells)	Incubator 3% O_2_	48 h	n.d.	Increase of c-Kyt positive cells (Most primitive and undifferentiated population of cardiac stem cells).	[148]
Mouse EG (Embrionic Germ cells) PGC (Primordial germ cells)	Incubator 3% O_2_	1 d, 3 d, 7 d	Protein detection (immunofluorescent Staining for HIF-1α)	Hypoxia exposure does not induce Klf4 or c-Myc upregulation in PGCs. Hypoxia promotes a metabolic switch from OxPhos toward glycolysis in PGCs and hypoxic PHCs showed reduced ROS levels vs normoxic ones.	[149]
human RPCs (Retinal Progenitor cells)	Incubator 3% O_2_	1, 5, 10 passages	Protein expression (HIF-1α and HIF-2α)	Increase of stemness markers expression, Oct-4, Sox-2, c-Myc, and Klf-4	[150]
mouse BMMSCs (Bone Marrow Mesenchymal stem cells)	Incubator 3% O_2_	7 d	n.d.	Increase of stemness markers expression, Oct-4 and Rex-1. 3% O_2_ augmented the amount of colony-forming cells by 1.6-fold vs. 21% O_2_.	[151]
human MSCs (Mesenchymal stem cells)	Incubator 3% O_2_	p0–p4 passages?	RNA expression (*HIF-1α*)	Hypoxia decreases differentiation potential and increases colony formations number. Under 20% the expression of Nanog and Rex-1 decreases and MSCs show a more senescent phenotype (evaluated with senescence associated markers expression).	[152]
human MIAMI cells (marrow-isolated adult multilineage inducible cells)	Incubator 3% O_2_	3 w	RNA expression (*HIF-1α*)	Increase of stemness markers expression, Oct-4 and Rex-1.	[153]
buffalo ASCs (Adipose derived stem cells)	Hypoxic chamber 5% O_2_	3 or 6 passages	RNA expression (*HIF-1α*)	Increase of stemness markers expression, Oct-4, Nanog, and c-Myc.	[154]
human RPCs (Retinal progenitor cells)	Incubator 5% O_2_ with or without KOSR (Knock-out serum replacement)	Until confluency	n.d.	Increase of stemness markers expression, c-Myc and Oct-4, in both conditions(With or without KOSR) vs. normoxia.	[155]
Urine stem cells (USC), Dental pulp stem cells (DPSC), Amniuotic fluid stem cells (AFSC), Bone Marrow stem cells (BMSCs)	Hypoxic chamber 5% O_2_	5 d	RNA expression (*HIF-1α*)	Increase of stemness markers expression (Oct-4, c-Myc, Nanog, and Nestin). Hypoxia showed also increased proliferation rate, inhibition of senescence, and increased differentiation ability.	[156]
human PMSCs (Placenta derived stem cells)	hypoxic chamber 5% O_2_	3 d	n.d.	Increase of stemness markers expression, Oct-4, Sox-2, and Nanog.	[157]
dog ADMSCs (Adipose derived Mesenchymal stem cells)	hypoxic chamber 5% O_2_	3rd passage	RNA expression (*HIF-1α*)	Increase of stemness markers expression, Oct-4, Sox-2, and Nanog,	[158]
human ESCs (Embryonic stem cells)	Incubator 5% O_2_	3 passages (followed by -reoxigenation)	RNA expression and ChIP analysis (*HIF-2α*)	HIF-2α enhances stemness by binding to an oct-sox cis-regulatory element in the promoter region of Nanog.	[159]
human WJMSCs (Wharton Jelly’s mesenchymal stem cells)	Incubator 5% O_2_	2–4 w	RNA expression (*HIF-1α* and *HIF-2α*) + immunodetection (HIF-1α)	Increase of stemness markers expression, Oct-4A, Sox-2, Rex-1, and Nanog.	[160]
mouse GSCs (Germline stem cells)	incubator 5% O_2_	7 d	Protein expression (HIF-1α and HIF-2α)	Increase of stemness markers expression, Oct-4, Sox-2, Klf-4, and Nanog.	[161]
human TSCs (Tendon stem cells)	Incubator 5% O_2_	3–5 d	n.d.	In cultures under 5% O_2_, more hTSCs expressed the stem cell markers nucleostemin, Oct-4, Nanog and SSEA-4.	[162]
human ESCs (Embryonic stem cells)	Incubator 5% O_2_	7–14 d	n.d.	No major difference in representative stemness genes (*Oct-3/4*, *Nanog*, and *Cripto*),	[163]
human TSCs (Tendon stem cells)	Incubator 0.5–5–10% O_2_	2 passages	n.d.	10% and 5% O_2_ increases stemness markers expression (5% has emerged as the optimal concentration) and both increases colony formation and number. 0.5% O_2_ decreases stemness.	[164]
mouse iHepSCs (Induced-hepatic stem cells)	Incubator 10% O_2_	6 h, 24 h, 72 h, 120 h	RNA and protein expression (HIF-1α and HIF-2α)	Enhanced stemness through faster progenitor proliferation rate; hypoxia accelerate G1/S transition through HIFs activation. Moreover, p53 and p21 in hypoxia-cultured iHepSCs were inhibited and CDKs (ciclin D kinase complexes) were upregulated.	[165]
mouse iSCs (ischemia induced stem cells) from brain ischemic regions	Ischemic areas/ cerebral infarction	-	n.d.	Cells from ischemic region, as well as control ES cells, exhibited pluripotency markers: c-Myc, Klf-4, Sox-2, and Nanog.	[53]
**Cell Source**	**Other**	**Time of** **Exposure**	**HIF Analysis**	**Hypoxic Effect on Stemness**	**Reference**
Human WJ-MSCs (Wharton’s Jelly MSCs)	None	-	RNA expression (*HIF-1α* and *HIF-2α*)	Gene expression related to the stemness properties showed differences according to ALDH activity. *HIF*s, *Glut-1* and stemness gene (*Oct-4, Nanog, and Rex-1*) are more expressed in ALDH+ positive cells compared to ALDH- cells.	[166]
Human AT-MSCs (Adipose Tissue MSCs)	None	-	RNA expression (*HIF-1α* and *HIF-2α*)	*HIFs, Glut-1*, and stemness genes (*Oct-4, Nanog,* and *Rex-1*) are more expressed in ALDH+ positive cells compared to ALDH- cells.	[167]
Human FSK-MSCs (Foreskin derived MSCs)	None	-	RNA expression (*HIF-1α* and *HIF-2α*)	*HIFs, Glut-1* and stemness gene (*Oct-4, Nanog* and *Rex-1*) are more expressed in ALDH+ positive cells compared to ALDH- cells.	[168]
Human BMMSCs (Bone marrow MSCs)	None	-	RNA expression (*HIF-1α* and *HIF-2α*)	*HIF* and stemness gene (especially *Oct-4* and *Rex-1*), *HIFs* and *Glut-1* are more expressed in ALDH+ positive cells compared to ALDH- cells.	[169]

## 4. Exploitation of Hypoxia for Regenerative Medicine Purposes

The potential application of stem cells for tissue regeneration represents an important challenge but it is necessary to optimize cell culture conditions to preserve the desired stem cell properties. As reported, some in vitro parameters can influence stem cell characteristics and reduce cellular proliferation supporting senescence [170]. One prominent characteristic of stem cells is their natural aptitude to reside in low O_2_ condition niches. Of note, efforts have been made in the last years to exploit the possibility to grow these cells in hypoxic conditions to mimic the naive microenvironment looking for valid in vitro culture protocols improving the stemness phenotype [171]. The regulation of self-renewal properties by O_2_ can also indirectly depend on HIF stabilization (in Section 1.3) as it represents a key determinant of the activation of stemness genes (such as *Oct-4*, *Sox-2*, or *Nanog*) and metabolic-related factors [172].

O_2_ can even directly regulate the stem cell fate since it is a cellular metabolic substrate and pluripotency is also characterized by specific metabolic and mitochondrial responses. Although most studies explain the hypoxia induction mechanisms related to stemness preservation especially in pluripotent cells, as ESCs or pluripotent cells (PSCs), several types of cells benefit from low O_2_ gradient for stemness retention as we further describe in Chapter 5. However, a briefly introduction on the role of stemness expression markers and metabolism in regulating stem cell fate and how hypoxia fits in stemness control are reported in the following sections.

### 4.1. Pluripotency-Related Markers of Stem Cells

Pluripotency requires the expression of important transcription factors such as *Oct-4*, *Sox-2*, and *Nanog* which are pivotal to orchestrate a complex interdependent transcriptional network in SCs [173] that might direct cell identity, as shown with genome-wide studies [174]. In addition, several proteins were identified as implicated in the control of cell self-renewal (Esrrb and Zfx), in the regulation of cell cycle progression (E2F1, c-Myc, and Klf4), and the maintenance of cell wellness (BMP-induced Smad1 and LIF) [124]. All these factors are also strictly correlated in their expression; for example, Nanog is an upstream regulator of the signal transducer and activator of transcription factor 3 (STAT3) and Oct-4, and its regulation of pluripotency and cell differentiation occurs through the interaction with a transcription factors complexes containing *Oct-4*, *Sox-2*, and *Klf4* [175]. Furthermore, Nanog and Oct-4 also can interact and co-occupy target genes of Nanog suggesting a cooperation of these transcription factors in the control of gene expression [175].

Although Sox-2, Oct-4, and Nanog are probably the most investigated stemness markers, Rex-1 is another factor which, in combination with the above-mentioned ones, plays a functional role in promoting cell cycle progression and stemness maintenance [176].

Together with genetic factors, epigenetic modifications contribute to create stem cells heterogeneity through different mechanisms such as DNA methylation, nucleosome remodeling, and post-translational modifications on histone tails generating a high variability of enhancers, conferring specific gene expression to cellular types [177].

Given that pluripotency is a dynamic state, the development and maintenance of stem cells is strictly dependent on a synergic regulation of these principal transcription factors expression, their epigenetic modifications and cellular localization [178].

### 4.2. Metabolic State of Stem Cells

**Figure 6:**
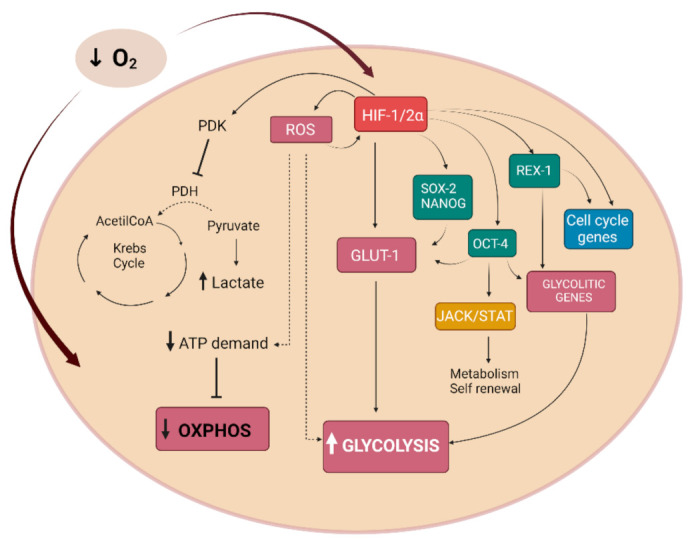
Summary of stem cell metabolic state during hypoxia condition and molecular pathways linking HIF-stemness genes-metabolism switch to glycolysis.

The metabolic state of stem cells is characterized by a specific profile represented with a dynamic mitochondrial morphology shift from glycolysis to mitochondrial oxidative phosphorylation (OxPhos) when cells pass to a more differentiated phenotype [179]. Therefore, the undifferentiated stem cells are characterized by glycolysis instead of oxidative phosphorylation [180] (Figure 6).

An elevated glycolytic flux is common to highly proliferating cell types, suggesting that this process has a central role in the acquisition and maintenance of pluripotency [181]. Self-renewing stem cells have significantly lower levels of mitochondrial activity, antioxidant enzymes, oxidative proteins, ROS levels, and lipid hydroperoxides [182]. So, manipulating metabolic pathways, with either genetic approaches or drugs or culture conditions, can directly affect whether stem cells remain quiescent, self-renew, or differentiate [183].

It has been described how ESCs and iPSCs, as totipotent stem cells, are characterized by high plasticity and can potentially be directed to any cell type. Certainly, the metabolic characteristics of iPSCs are not completely the same as ESCs, but it is not a detail that both primarily rely on glycolysis to meet energy requirements, in contrast to their somatic counterparts [184]. The set of evidence that correlate “stemness” and “metabolism” has driven the development of new systems that can be adopted to generate iPSC relying on small molecules that enhances cellular reprogramming through the promotion of glycolytic metabolism [185]. Coherently with this data, it has been reported that fibroblasts preconditioning with culture medium containing lactate resulted in a switch from OxPhos to glycolysis, in part, through ROS-mediated stabilization of HIF-1α [186]. Interestingly, reverting cells to an immature state, required the expression of factors involved in mitochondrial biogenesis, morphology and distribution, intracellular ATP production, and lactate generation, supporting the role of the metabolic state in stem cell fate [184]. Consistent with this aspect, the transcription factors involved in stemness preservation, such as Oct-4, also take part in the metabolic control as the key reprogramming factor Oct-4 has been shown to target multiple metabolic genes [187]. It has been demonstrated for example that in developing mice embryos, Oct-4 activate the JAK/STAT signaling pathway thus regulating cellular metabolic properties via energy metabolism, cell morphology, and chromatin accessibility [187]. Moreover, in mouse embryonic fibroblasts (MEFs), Rex-1 stimulates the expression of glycolytic genes, through the cyclin B activation, promoting glycolysis instead of OxPhos [176]. Glycolysis can be maintained by a constant glucose uptake by the glucose transporter 1 (Glut1) whose levels are finely regulated in hESCs [188]. The optimal levels of Glut1 expression are supported by the enhancer element of Glut1 (GE) which is accessible for the pluripotency factors Sox-2, Oct-4, and Nanog which can each bind to GE thus inducing the expression of Glut1 [188].

As mentioned above, the correlation between self-renewal properties and cellular metabolism is evidenced also in different type of multipotent stem cells. For example, no completely differentiated HSCs exhibit fewer mitochondria and higher glycolytic capacity in whole bone marrow [184]. Indeed, the levels of antioxidant enzymes, such as superoxide dismutase (SOD), are higher in circulating progenitor cells than in long-term quiescent HSCs which exhibits enhanced glycolytic status within mitochondrial activity and ROS balance cooperating and finely regulating self-renewal of these stem cells [184].

Moreover, in undifferentiated MSCs too, mitochondrial activities are maintained at low level while, at the same time, glycolytic activities are consistently maintained at high levels. Here, the glycolytic process contributes to greater than 97% of ATP production, in the energy metabolism of undifferentiated bone marrow MSCs [189]. The rapid uptake of glucose in MSC cultures confirms their dependence on glycolysis [190], and, moreover, undifferentiated MSCs produce high levels of lactate, suggesting a reliance on non-aerobic glycolysis to cover the bioenergetic needs [191].

The set of evidence reported in the literature supports the concept that cellular metabolism does not just represent only an energetic state but plays a central role in the determination of stem cell fate.

Finally, the preferential utilization of glycolysis over mitochondrial oxidative metabolism may also represent a mechanism to preserve the genomic integrity through the reduction of ROS production by OxPhos mechanism. Due to the ROS reduction, cells can limit possible damages within nuclear and mitochondrial DNA and even reduce the oxidation of proteins and lipids [192].

Although ROS have been considered just a metabolic waste product, in the past decades accumulating scientific evidence demonstrated their key role in cell fate signaling (Figure 6).

The acronym ROS refers to O_2_ reactive species, but it may also include several nitrogen-containing compounds reactive nitrogen species (RNS), such as nitroxyl anion (NO^−^), peroxynitrite (ONOO^−^), and nitric oxide (NO) which are produced by the activity of inducible nitric oxide synthase (iNOS) and reacts with superoxide to give rise to the other RNS [193].

Emerging evidence has demonstrated how modulation of ROS level and metabolic flux has a key role in dedifferentiation processes, as reported for the generation of iPSCs from differentiated cells that benefits from careful regulation of ROS levels [194].

Metabolism can affect signaling pathways through the modulation of ROS levels which can react with various proteins, such as kinases, phosphatases, or transcription factors, to alter processes linked to cell cycle progression, quiescence, or differentiation [195]. In turn, ROS can also regulate metabolic processes such as glycolysis, OxPhos, pentose phosphate pathway activity, and autophagy [196]. This complex metabolic regulation can also occur through the triggering of HIF accumulation which, in a positive feedback loop, stabilizes ROS and enhances Glut1 expression, promoting the metabolic switch in favor of glycolysis [196].

However, ROS levels must be tightly regulated to preserve cellular senescence and proliferation while avoiding a dysregulated ROS production that occurs in pathological conditions [197]. The importance of ROS homeostasis is evidenced in ESCs that present mechanisms for enhanced ROS removal capacity as well as limited ROS production, despite this cell type possess a limited number of mitochondria. Accordingly, a recent study reveals that the human iPSC generation process can effectively reduce the mitochondrial genome copy number and cells have similar ROS levels and antioxidant defenses to those seen in ESCs [198].

Consistently, higher mitochondrial activity and oxidative stress were found as one of the major causes of functional decline in stemness features [184]. Overall, ROS should be considered as signaling molecules orchestrating the crosstalk between metabolism and stem cell fate decisions.

### 4.3. O_2_ for Stemness Preservation

Self-renewal and potency hallmarks of stem cells are influenced by several intrinsic and extrinsic cell factors. As previously reported low O_2_ concentration, hypoxia, has been closely linked to the maintenance of stemness. For most cell types, hypoxia acts as a modulator of cell proliferation decreasing the levels of respiratory enzymes meanwhile increasing the production of glycolytic enzymes and lactate, thus enhancing the glycolysis process [179]. In details, hypoxic conditions reduce the availability of molecular O_2_ suppressing the activity of the mitochondrial electron transport chain. Cells switch to glycolysis also through HIFs activity, reducing the expression of mitochondrial enzymes and further enhancing the shift to glycolysis by upregulating glucose transporters and glycolytic enzymes [179].

The limited availability of molecular O_2_ under hypoxic conditions results in the reduction of the mitochondrial electron transport chain (ETC) activity and cells switch from OxPhos to glycolysis to reach their energetic needs, since it does not require O_2_ [199]. On the other hand, in presence of abundant O_2_ levels, pyruvate dehydrogenase (PDH) converts pyruvate produced from glycolysis to acetyl coenzyme A (Acetyl-CoA) giving start to the tricarboxylic acid cycle (TCA cycle). This process is regulated by the pyruvate dehydrogenase kinase (PDK) that phosphorylates and inactivates PDH, thus playing a crucial role in metabolic adaptation of cell in response to hypoxia and it is transcriptionally regulated by HIF-α [200].

In addition, PDK attenuates mitochondrial ROS production, which is critical as increases in glycolytic flux can be associated with leakage of electrons from the respiratory chain resulting in unexpectedly elevated ROS levels [201].

Because high O_2_ levels contribute to a decline in stem cell properties, low O_2_ pressure should reflect the better physiological conditions for the cells and this aspect must be considered when culturing them. Stem cells cultured under hypoxic conditions can enhance their self-renewal ability and retain their pluripotent capacity, as it has been demonstrated in MSCs or HSCs [7]. In a low O_2_ culture, MSCs improve the maintenance of their undifferentiated state through the suppression of mitochondrial activity and promote genetic stability [202]. Even more, adult HSCs, residing in low O_2_ niches, have a metabolism that is mainly based on glycolysis for the energy demand, and an increase in mitochondrial activity is linked to a decline in stemness [203].

In literature, there are evidence supporting complex link connecting hypoxia, metabolism, HIFs, and several molecules with crucial roles in the regulation of stemness or differentiation (Figure 6). Low O_2_ tension can upregulate proliferation and the expression of pluripotency-related genes, probably by mimicking the conditions that stem cells experience in vivo [170]. In turn, these stemness factors can regulate other subordinate genes involved in the metabolic control of stemness [204] allowing the preservation of a more undifferentiated state and genetic stability (Figure 6).

### 4.4. HIF Role in Stemness

It has been described that stem cells exist in physiological low O_2_ environments with a metabolism that relies on glycolysis instead of oxidative phosphorylation [180], and that hypoxia can regulate stem cell plasticity through the action of HIFs. In the HSC stem cell model, it was demonstrated that HIF-1α stabilization is correlated to the maintenance of an anaerobic metabolism through the transcriptional activation of genes regulating glycolysis, such as *GLUT1* and *PDK1* [205,206], and transcriptional inactivation of genes encoding for key mitochondrial electron transport chain enzymes e.g., phosphoglycerate kinase-1 (*PGK1*), or lactate dehydrogenase-A (*LDHA*), and glucose transporters (such as *GLUT1* and *GLUT3*) [200] (Figure 2 and Figure 6).

HIF-1α can regulate the HSCs metabolism after the transcriptional activation by the homeobox transcription factor myeloid ectotrophic viral integration site 1 (*Meis1*) [207], which is important also for transcriptional activation of HIF-2α. The loss of Meis1 in HSCs results in disruption of stem cells quiescence through increased ROS production, increased apoptosis and down-regulation of both HIF-1α and HIF-2α [208]. Thus, HIF-1α and ROS closely work together, along with O_2_ homeostasis and energy metabolism, to maintain HSCs function [203].

HIF role in the metabolic status of stem cells can also affect stem cells immunoregulatory properties. HIF-1α silencing in MSCs resulted in affected capability to reduce inflammation and inhibit the generation of pro-inflammatory T cells. This impaired immunosuppressive potential was correlated to the metabolic switch from glycolysis to oxidative phosphorylation and a reduced ability to produce immunosuppressive mediators such as intercellular adhesion molecule (ICAM), interleukin-6 (IL-6), and nitric oxide (NO) [209].

HIF can preserve stemness also preventing apoptosis through the downregulation of p53 involved in cell survival [210]. It was demonstrated that HIF-1α overexpression, induced by transfection, suppressed p53, the downstream factors p21, and increased B-cell lymphoma 2 (BCL2) [211], an anti-apoptotic factor that can be suppressed by p53 [212]. On the other hand, p53 can induce the transcriptional activation of p21 that participates in apoptotic regulation modulating the cell cycle [213].

HIF capability to preserve stemness could be even correlated with aldehyde dehydrogenase (ALDH) activity as observed in Wharton’s Jelly stem cells [166]. Adipose-derived stem cells [167], foreskin-derived mesenchymal stromal cells [168], and for bone marrow mesenchymal stromal cell [169]. ALDHs are enzymes responsible for the oxidation of aldehydes to their corresponding carboxylic acids. The main role of ALDHs is the catalysis of aldehydes [214] that can be toxic for the body. ALDHs are also involved in cell proliferation, embryogenesis, development, cell signaling, neurotransmission, protection from UV radiations, drug metabolism, osmoregulation, gene regulation, and redox balance [215,216]. It was shown that ALDH^+^ sorted stem cells displayed a major hypoxia response compared to ALDH^−^ stem cells, increasing of HIF-1α and HIF-2α. Moreover, ALDH^+^ stem cells exhibited an increased mRNA expression of stemness correlated genes *Oct-4*, *Nanog*, *Sox-2*, and *Rex-1* compared to ALDH^-^ cells [166].

HIF-1α downregulation by UVA irradiation was responsible for the decrease of MSC stemness properties. This effect was due to the upregulation of prostaglandin E2 (PGE2) and its downstream molecule, cyclic adenosine monophosphate (cAMP), through the activations of activator protein 1 (AP-1) and nuclear factor kappa-light-chain-enhancer of activated B cells (NF-κB) [98,99,100,101]. The cAMP molecule can reduce the expression of HIF-1α gene through the cAMP response element-binding protein (CREB) activation, downregulating the expression of the stemness genes *Nanog*, *Sox-2*, and *Oct-4*. However, some chemical compounds can attenuate the UVA-induced effects on the expression of the stemness genes by inhibiting p38 MAPK and NF-κB, the upstream factors in the PGE2 production, such as the arctigenin [101], sinapic acid [100], aspartic acid [99], or ethylcysteine [98].

In this regard, it has been demonstrated that HIF-1α is involved in a pathway that influences MSCs proliferation and migration [217]. In particular, hypoxia stimulates UCB-hMSC proliferation through the expression of the fatty acid synthase (FASN) and stearoyl-CoA desaturase-1 (SCD1), two lipogenic enzymes, whose expression was regulated by the HIF-1α/SCAP/SREBP1 pathway. This pathway was able to also induce the phosphorylation of the mammalian target of rapamycin (mTOR) [217] as CDK2, CDK4, cyclin D1, cyclin E, and F-actin expression as well as c-Myc, p-cofilin, profilin, and Rho GTPase, involved in cells cycle [217]. Moreover, stem cell proliferation was also related to HIF-1 phosphorylation, and other different substrate, by phosphatidylinositol 3-kinase (PI3K)/protein kinase B (Akt) signaling pathway activation [147]. Interestingly stemness can be influenced by HIF-2α as it targets specifically Oct-4 [218] that synergically cooperate with Nanog and Sox-2 to maintain stem cells properties and avoid differentiation [219]. Indeed, it was also demonstrated that HIF-2α expression preserved human placenta-derived mesenchymal stem cells (hPMSC) stemness and promoted their proliferation by regulating CyclinD1 (CCND1), c-Myc, and Oct-4 through the MAPK/ERK signaling pathway [120].

However, despite the increasing evidence reported in the literature about the link between stem cell plasticity and HIFs, the exact molecular mechanisms through which HIF influences stem cells preservation are not completely elucidated yet, due to the complexity and multiple crosstalk of signal pathways involved.

## 5. Hypoxia Cells Models

Hypoxia maintains a slow-cycling proliferation property, reduced oxidative stress, and undifferentiated status in several stem cell populations [220]. Different cellular models have a peculiar way to adapt to O_2_ availability probably due to the different naïve niches. Indeed, cells derived from different tissues have different O_2_ consumption rate and O_2_ concentration can influence their behavior in vitro. Due to the importance to optimize protocols for preserving stemness properties of cultured cells and preventing senescent phenotype that occurs after a long period in culture, in this chapter it has been considered the wide stem cells models (embryonal, fetal, and adult stem cells) studied with different in vitro hypoxia approaches using different O_2_ concentration. Table 3 summarized the principal results data specifically focusing the attention of hypoxic effects on growth and maintenance of pluripotency and differentiation.

Embryonal stem cells (ESCs). A growing number of studies confirm that hypoxia plays a role in the human ESCs niche through metabolic shifts and HIF regulatory elements [221]. Accordingly, the protein interaction (PPI) analysis performed by Murugesan and Premkumar [124] indicates specific genes with distinct roles in the regulation of metabolic shift contributing to hypoxic mediated stem cell niche. Moreover, low O_2_ tension conditions could reduce the amount of spontaneous cell differentiation that normally occurs in hESCs in vitro, appearing as an important element that can help to maintain cells in a fully pluripotent state [222]. Therefore, it is not completely evident if reduced O_2_ tensions are mandatory for the maintenance of full pluripotency. In vivo, inner cell mass normally undergoes differentiation, and therefore, it could be supposed that the maintenance of hESCs in an undifferentiated state does not represent a physiologic condition. This suggests that low O_2_ culture may be optimal if the aim is to differentiate the hESCs thus reproducing the physiologic condition of embryo growth in vivo, whereas normoxic cultures may be better for the maintenance of hESCs in an undifferentiated state since hESCs naturally tend to differentiate [223] (Table 3).

Fetal stem cells (FSCs). A promising category of stem cell is now represented by FSCs cells that can be isolated from placenta and extraembryonic tissues. Their intermediate state between adult and embryonic stem cells makes them an ideal candidate to be used for regenerative medicine. Many multipotent stem cells have been isolated from different parts of the placenta, placenta-derived MSCs (PMSCs), and, even, from the amnion, chorion, umbilical cord, and fetal blood [224]. Amnion-derived stem cells can include amniotic epithelial stem cells (AECs), amniotic fluid stem cells (AFCs), and amniotic mesenchymal stem cells (AMCs).

Up to date few studies have focused on the hypoxic PMSCs although the first trimester of human placental occurs in a low O_2_ environment, 2–3% O_2_ percentage that can even promote stemness and proliferation of the trophoblast lineage stem cells (TSCs), the progenitors of placenta stem cells. These cells appear to be very vulnerable to high O_2_ concentration indeed the choice of the proper O_2_ physiological levels for TSCs cultures is a crucial aspect [70].

Amniotic stem cells (AECs) have received great interest because of their availability and their multilineage differentiation potential in vitro [225,226] and innate low immunogenicity [227,228] that make cells ideal candidates for tissue repair [229]. AECs, as well as umbilical cord-derived MSCs isolated from the perivascular region of the umbilical cord (HUCPVCs) or Wharton’s jelly (WJ-MSCs) positively respond to hypoxia in favor of both stemness retention and differentiation (Table 3).

Adult stem cells. In HSCs or MSCs, hypoxia appears to prolong the lifespan of the stem cells, increases their proliferative capacity, and reduces differentiation in culture. A high level of HIF-1α expression was found in the primitive HSCs, which decreases as differentiation progresses and regulates several glycolytic enzymes that are under the control of HIF. Moreover, the primitive HSCs were low O_2_ consuming and show high glycolytic flux and lower mitochondrial mass. Quiescent HSCs show low mitochondrial potential to avoid oxidative stress and ROS accumulation leading to decreased stemness and spontaneous differentiation [203]. Certainly, MSCs are the best-characterized stem cell type in hypoxic culture conditions. MSCs derived from several tissues, such as chondrocytes, adipocytes, osteocytes, bone marrow, tooth, and amniotic fluid, in hypoxic culture conditions expressed higher levels of stemness markers as *Oct-4*, *c-Myc*, *Nanog*, and *Nestin*. Moreover, hypoxia was able to inhibit senescence and increase the proliferation rate and differentiation ability [156]. Nevertheless, from literature data emerged that the different outcomes of MSC were strictly dependent on the O_2_ concentration and the intrinsic properties of cell types. Indeed, in the study of Wagner et al., it was reported a list of MSC types with their respective O_2_ consumption rates (OCRs) indicating that, in this category of cells, the redox environment depends on the levels of antioxidant and redox enzymes which influence cellular outcomes [63]. Therefore, MSCs take great advantage of hypoxic cultures, resulting in an advantageous condition to preserve stemness and differentiation potential for a long period in vitro.

**Table 3:** Summarized hypoxia responses of mostly used stem cell sources in in vitro hypoxia cultures.

Stem Cell Source	Hypoxia Effects	Reference
Embryonic stem cells (ESCs)	5% O_2_ tension did not change hESCs morphology” on day 7 of the first and fourth passages. After 10–14 days, hESC colonies were thinner and looked better morphologically in 5% O_2_, but cells’ proliferation was slower, and their sizes were larger. No significant differences in representative stemness genes (*Oct-3/4*, *Nanog* and *Cripto*), differentiation genes (*Desmin*, *α-fetoprotein* and *GDF-9*), and hypoxia-related genes *HIF-1α* and *VEGF*. The short-term stabilization of HIF, mediated by 1% or 5% hypoxia and PHD inhibitors, do not prevent the spontaneous loss of pluripotency of mouse ESCs naturally differentiating. In 1% O_2_ condition, mESCs started to differentiate losing their self-renewal ability through the downregulation of H2afz gene that has been identified as new potential target gene involved in the maintenance of pluripotency in ESCs	[92,134,163]
Placental stem cells (PMSCs)	5% O_2_ culture not altered morphology of PMSCS, while the expression of pluripotency markers Oct-4, Nanog, and Sox-2 is increased. In 5% O_2_ exhibited a more naïve morphology and had a higher proliferative capability and higher HIF-2α expression than hPMSCs cultured in 21% O_2._ PMSCs over-expressing HIF-2α showed higher proliferative potential and higher expression of CCND1 (CyclinD1), c-Myc, Oct-4 and components of the MAPK/ERK pathway. In contrast, these genes were down-regulated in the HIF-2α-silenced hPMSCs.	[120,157]
Amniotic Epithelial stem cells (AECs), Amniotic Fluid stem cells (AFCs), and Wharton’s Jelly MSCs (WJ-MSC)	AECs positively respond to in vitro hypoxia (1% O_2_) as combined islet-cell (IC) and hAECs organoids, cultured under hypoxic conditions, showed considerable protection from cell death, under ischemic stress. This protective effect of hAECs on islet cells can be linked to HIF-1α that orchestrate compensatory responses to hypoxia. 2% O_2_ can be a suitable culture condition to induce tenogenesis in AECs as demonstrated by upregulation of tenomodulin in AECs. This suggest that hypoxic culture could be beneficial even for tendon structure formation. After low O_2_ conditioning (1% O_2_), the secretome of AFCs augments cardiomyocyte proliferation enhancing cardioprotective effects under hypoxic-ischemic conditions proving that O_2_ modulation can be an efficient physical cue to produce secretomes enriched in soluble bioactive factors helpful for cardiac applications. 5% O_2_ rejuvenates WJ-MSCs which appear less-differentiated, more primitive and faster-growing involving HIF-1α and HIF-2α. In correlation with the up-regulation of HIF-1α and HIF-2α there is an increase expression of stemness marker Oct-4, a direct down-stream target of HIF. Even 3% O_2_ is a favorable concentration, enhancing WJ-MSCs clonogenicity and expansion capacity and stemness markers expression.	[146,160,230,231,232]
Trophoblast stem cells (TSCs)	0.5% O_2_ exposure caused a reduction of ERB2 and ID2 expression, specific trophoblast stemness markers, in mouse TSCs, and favored differentiation while, under 2% O_2_, the same cells maintained their potency.	[70,122]
iHepSCs	iHepSCs lost their stemness features and presented a diminished cellular expansion in 1% O_2_ culture while 10% O_2_ enhanced stemness through cell progenitor proliferation in iHepSCs appearing as the optimal concentration.	[165]
Hematopoietic stem cells (HSCs)	Low O_2_ tension plays a crucial role in maintaining normal HSC function protecting cells from differentiation or senescence through the hypoxia-Fbxw7 pathway.	[233]
Bone Marrow derived MSCs (BM-MSCs).	1% O_2_ tension culture determined a delayed progression of cellular senescence through the activation of the serine/threonine kinase AKT pathway; improved the proliferation rate and increased the expression of stemness genes such as Oct-4, Klf4, and Nanog. 1% O_2_, through HIF-1α activation, activated the Notch-2-c-myc pathway that is required for the inhibition of senescence and proliferation promotion in mouse BM-MSCs.Under 3% O_2_, mouse BMMSCs cultures, exhibited a significative increase of proliferation rate and augmented colony formation number.	[127,129,130,133,151]
Adipose derived MSCs (AMSCs or ASCs).	A range from 1 to 5% O_2_ seems to preserve stemness of AMSCs. These cells clearly show a differentiation potential towards the cell type of the mesoderm lineage (adipocytes, chondrocytes, and osteoblasts) right from the early passages in culture. The greater ability to preserve the stemness of hASCs is indicated by the increased expression of stemness genes *Nanog, Sox-2*, *Oct-4*, and *Rex-1*. It has been demonstrated that the upregulation of HIF-1α in hASCs in turn activates the downstream target genes resulting in a significantly favored cell proliferation and in the preservation of stemness genes expression.	[131,135,138,145]
Dental pulp stem cells (DPCs), Periodontal ligament cells (PDLCs), and stem cells from human exfoliated deciduous teeth (SHEDs).	2% O_2_ favored stemness maintenance with enhanced expression of Oct-4, Sox-2, and c-Myc in PDLCs and DPCs while they inevitably undergo to replicative senescence under current culture conditions (21% O_2_), resulting in cellular phenotypic changes. 3% O_2_ showed PI3K/Akt pathway activation and inhibition of oxidative stress in a ROS-dependent manner suggesting that regulation of self-renewal in DPCs may involve ROS control. Cobalt Chloride treatment promoted stemness retention, in a dose dependent manner, in DPCs, PDLSC, and SHEDs.	[105,114,115,117,139,147]
Tendon stem cells (TSCs)	O_2_ culture at 5% O_2_ of human TSCs enhanced cell proliferation. The expression of stem cell marker genes, Nanog and Oct-4, was upregulated. After in vivo implantation, more tendon-like structures were formed in the 5% O_2_. TSCs although deriving from a considerable hypoxic niche, showed a reduction of stemness markers expression when cultured under 0.5% O_2_ while 10% O_2_ and 5% O_2_ preserved their stemness; in particular, 5% O_2_ appeared to be the best concentration for the increase of stemness markers expression.	[162,164]
Cardiac progenitor cells (CPCs) and Cardiosphere derived cells (CDCs).	O_2_ CPCs expanded in 5% O_2_ increased cell yield, showed lower senescence and higher resistance to oxidative stress than those grown in 20% O_2_. In vivo implantation of cells grown in 5% O_2_ into mice infarcted hearts resulted in greater cell engraftment and better functional recovery. 3% O_2_ or chemical compounds- preconditioning of cardio-spheres derived cells cultures improved stemness properties of CDCs which presented a higher expression of HIF, pluripotency gene markers, and a significantly higher number of c-kit-positive cells which are the most primitive undifferentiated population of cardiac stem cells. Hypoxia-preconditioned CDCs exhibited decreased O_2_ consumption and improved glycolytic metabolism.	[143,148,234]
Neural stem cells (NSCs)	In 2D (neuroepithelial cell line (NECs) and embryonic neural tissue) and 3D (ESCs-derived neuro-spheres (NSCs) cellular model, it was demonstrated that HIF-1α endogenous stabilization preserve stemness in a hypoxic environment prevented the premature neuronal differentiation through the direct activation of the neural repressor hairy and enhancer of split 1 (Hes1) pathway.	[104]
Retinal progenitor cells (RPCs)	3% O_2_ to increases the proliferation rate of hRPCs shifting their proliferation limit. This increased proliferation was correlated with an upregulation of Ki67, CyclinD1, and telomerase activity and a decrease in p53 expression and apoptosis. Moreover, the increased c-Myc, Klf4, Oct-4, and Sox-2 expressions correlated with stabilization of both HIF-1α and HIF-2α was also detected in cells exposed to this hypoxic condition.	[155]

## 6. Hypoxia, Aging, and Stem Cell Transplantation

In the last part of this review, a short section is dedicated to the link between hypoxia, aging, and effects of stem cell transplantation for the treatment of tissue injury or disease, as O_2_ is a relevant factor even in the regulation of cell senescence.

When damage accumulates, mitotic cells from renewable tissues have two mechanisms to avoid replication. They can stop cell cycle progression and enter senescence or trigger cell death programs such as apoptosis. It is still unclear what determines if a cell undergoes senescence or apoptosis. Although most cells are capable of both, these processes seem to be exclusively yet linked to each other [7]. Inside tissues, O_2_ gradients exist with stem cells residing in their hypoxic niches. These cells, more resistant to oxidative stress as a mechanism of self-preservation, benefit from their hypoxic environments by avoiding senescence, which would be detrimental to the tissue regenerative capacity. However, the tissues of a multicellular organism inevitably go toward a decline in organ function with aging. Although adult stem cells can self-renew and differentiate into multiple cell types within a tissue, they are not immune to damage accumulation over time [235]. Beyond the accumulation in DNA damage, mutations, and epigenetic alterations, the aging of the stem cell environment can also seriously alter stem cell functions even resulting in the niche deterioration [235]. Another hallmark of cellular aging is given by the shortening of telomerase with age [236]. Indeed, cellular senescence naturally occurs because of the gradual shortening of telomeres deriving from continuous replication [237]. Interestingly, it has been demonstrated that hypoxia can extend cell lifespan through the upregulation of the telomerase activity, reducing the senescent response. Of note, the telomerase reverse transcriptase (TERT) is another target of HIF-1α [238]. The telomere length is directly correlated to the age of the donor, to the time of culture before the senescence, and cell type. Slight differences exist in MSCs derived from Wharton jelly and those from the bone marrow regarding their phenotype, the telomerase activity, and the clonogenicity abilities after hypoxia or normoxia culture conditions, respectively [239]. These observations support the current view that MSCs properties are impacted by the tissue origin, especially if they derive from extraembryonic tissue or adult sources [240]. Importantly, as reported in the paper of Merini et al., [240] is strongly recommend that all biological issues related to the use of MSCs, such as the impact of tissue hypoxia, inflammation, and infection, should be well monitored to accelerate the transition from the bench to clinic [240]. Highlighting these features may improve the quality, safety, and efficiency of the future therapy.

Moreover, induction of senescence can be accelerated and prematurely induced by other environmental cues, including excessive oxidative stress [241]. Metabolism damage related to ROS increase is also a fundamental component in the aging process. In this context, as described above, hypoxia preconditioning prior to transplant, can effectively reduce ROS production in adult stem cells and improve their therapeutic efficiency in several in vivo ischemia or other disease models [242]. When cells are transplanted in the body, they face hypoxic in vivo environmental conditions, and a significant number of grafted cells die because of the severe in vivo environmental conditions at transplanted site [242]. The cell death due to hypoxic microenvironment is particularly considerable for those tissues that are not vascularized or already injured [243]. For example, in heart transplantation, donor hearts inevitably suffer from ischemia/reperfusion (I/R) injury, which leads to primary graft dysfunctions and affects patient survival rate. Remarkably, hypoxic conditioned medium derived from BMSCs enhances post-transplant graft functions, via paracrine effects that are improved by the hypoxic culture conditions [244].

Although these are encouraging reports, a consideration that deserves to be deeper investigated is whether replicative senescence limits the therapeutic potential of stem cells.

Nonetheless, the transplantation success of young or rejuvenated stem cells in aged patients is still problematic, since stem cell function is greatly influenced by extrinsic factors that become unsupportive with aging [245]. Confirming this, MSCs from aged donors did not perform as well as cells from younger donors in a transplantation following myocardial infarction [246] and similarly, MSCs obtained from young individuals have been induced to neuroectodermal differentiation in vitro, but this effect cannot be replicated in MSCs from elderly individuals [247].

Furthermore, hypoxia preconditioned BMSCs with up-regulated HIF-1α can enhance the bone healing process in geriatric individuals [248]. More in detail, the combination of hypoxia and DMOG preconditioning significantly increased the survival rate in bone defect site of transplanted BMSCs and may have great potential in regenerative cell therapy for bone defects in aged individuals [248].

Together, these data suggest that the use of stem cells from young donors or the rejuvenation of aged patient-derived stem cells may represent a promising system to improve the efficiency of transplantation. The preconditioning of MSCs in hypoxia triggers, even via the stabilization of HIF-1α, the upregulation of different functions, helping MSCs to survive after implantation, and increasing their curative potential [249]. O_2_, in a range between 1% and 5%, is a low concentration, adequate to trigger adaptation, but not excessively low to cause apoptosis [250]. However, exact details of hypoxic treatment protocols (O_2_ concentration, time of preconditioning, isolation under hypoxia, and reoxygenation) are still under examination to achieve a successful protocols optimization.

## 7. Conclusions

The present review aims to highlight the correlation existing between hypoxia and stemness focusing on cell culture models as invaluable research instruments for the comprehension of physiological hypoxia-induced mechanisms enabling the development of novel approaches to improve stem cell-based therapeutic strategies.

The O_2_ tension, lowered to mimic niche microenvironment, has been successfully proposed to preserve cells phenotype during expansion for stem cell populations limited in supply. Alternatively, hypoxia has been adopted in vitro as a valuable stimulus to promote cell commitment into different tissue lineages. Beside this encouraging evidence, most of the protocols validated to date to expand stem cells recognize 21% O_2_ tension (air O_2_ concentration), which is about from 4- to 10-fold greater than gas levels in the natural niches by exposing the cells to cultural conditions that enhance oxidative stress and change cell metabolism with unpredictable and deleterious effects on stem cells phenotype and fate. Evidence emerged from available literature, mainly demonstrated a large spectrum of low O_2_ in vitro effects on stemness maintenance, cell proliferation, senescence inhibition, and cell plasticity [137,156]. The hypoxic conditions reproduced in culture are mostly obtained by lowering O_2_ tension in a range from 1% to 5% while levels lower than 1% are perceived from several cells as anoxic ones.

However, accurate control of O_2_ levels has been suggested as a prerequisite to improving the reproducibility of the results as well as to compare them amongst laboratories by considering that small changes in pericellular O_2_ levels can elicit profound molecular and signaling intracellular responses. To this aim, the use of O_2_ sensors is considered a value approach to have a real-time O_2_ monitoring under in vitro culture. Recently, several accessible, cost-effective, and high-throughput tools able to emulate controlled hypoxic conditions reproducing a steady or intermittent exposure closely mimicking the in vivo conditions have made available. Alternatively, hypoxia is induced by adopting chemical approaches that have been proposed taking advantage of their great flexibility and reproducibility in studying the acute effect of hypoxia.

In addition, higher levels of standardization of the in vitro conditions would be beneficial to interpret and compare the hypoxia-mediated HIF activation and stem cell response in term of hypoxic inductive physical or chemical methods (i.e., O_2_ tension or drug concentrations), stem cell source, cultural parameters (cell concentration, degree of confluence, medium, and supplements), and time of hypoxic exposure (acute and chronic).

Of note, O_2_ has been successfully suggested to provide a precommitment of stem cells before their therapeutic use. Indeed, the hypoxic imprinting of stem cells is an emerging in vitro strategy to improve tissue regeneration [251,252], strongly suggesting that decoding the mechanisms, by which cells sense O_2_, could be useful for the development of new target molecules and stem cell-based treatment for several diseases, including cancer, stroke, and inflammation. Not by chance, in 2019, a trio of researchers, Gregg Semenza, William Kaelin, and Peter Ratcliffe, received the Nobel Prize in Physiology or Medicine for their discoveries on how cells sense and adapt to O_2_ variations thus opening new cell biology paradigms recognizing the central role that O_2_ may have in controlling cell response and adaptation.
